# Altered gut metabolites and microbiota interactions are implicated in colorectal carcinogenesis and can be non-invasive diagnostic biomarkers

**DOI:** 10.1186/s40168-021-01208-5

**Published:** 2022-02-21

**Authors:** Olabisi Oluwabukola Coker, Changan Liu, William Ka Kei Wu, Sunny Hei Wong, Wei Jia, Joseph J. Y. Sung, Jun Yu

**Affiliations:** 1 State Key Laboratory of Digestive Disease, Li Ka Shing Institute of Health Sciences, CUHK Shenzhen Research Institute, The Chinese University of Hong Kong, Shatin, Hong Kong, China; 2 Department of Medicine and Therapeutics, The Chinese University of Hong Kong, Shatin, NT Hong Kong, China; 3 Department of Anaesthesia and Intensive Care, The Chinese University of Hong Kong, Shatin, Hong Kong, China; 4 School of Chinese Medicine, Hong Kong Baptist University, Kowloon Tong, Hong Kong, China; 5 Lee Kong Chian School of Medicine, Nanyang Technology University, Singapore, Singapore

**Keywords:** Stool metabolites, Gut microbiota, Colorectal adenoma, Biomarker

## Abstract

**Background:**

Gut microbiota contributes to colorectal cancer (CRC) pathogenesis through microbes and their metabolites. The importance of microbiota-associated metabolites in colorectal carcinogenesis highlights the need to investigate the gut metabolome along the adenoma-carcinoma sequence to determine their mechanistic implications in the pathogenesis of CRC. To date, how and which microbes and metabolites interactively promote early events of CRC development are still largely unclear. We aim to determine gut microbiota-associated metabolites and their linkage to colorectal carcinogenesis.

**Results:**

We performed metabolomics and metagenomics profiling on fecal samples from 386 subjects including 118 CRC patients, 140 colorectal adenomas (CRA) patients and 128 healthy subjects as normal controls (NC). We identified differences in the gut metabolite profiles among NC, CRA and CRC groups by partial least squares-discriminant and principal component analyses. Among the altered metabolites, norvaline and myristic acid showed increasing trends from NC, through CRA, to CRC. CRC-associated metabolites were enriched in branched-chain amino acids, aromatic amino acids and aminoacyl-tRNA biosynthesis pathways. Moreover, metabolites marker signature (twenty metabolites) classified CRC from NC subjects with an area under the curve (AUC) of 0.80, and CRC from CRA with an AUC of 0.79. Integrative analyses of metabolomics and metagenomics profiles demonstrated that the relationships among CRC-associated metabolites and bacteria were altered across CRC stages; certain associations exhibited increasing or decreasing strengths while some were reversed from negative to positive or vice versa. Combinations of gut bacteria with the metabolite markers improved their diagnostic performances; CRC vs NC, AUC: 0.94; CRC vs CRA, AUC 0.92; and CRA vs NC, AUC: 0.86, indicating a potential for early diagnosis of colorectal neoplasia.

**Conclusions:**

This study underscores potential early-driver metabolites in stages of colorectal tumorigenesis. The Integrated metabolite and microbiome analysis demonstrates that gut metabolites and their association with gut microbiota are perturbed along colorectal carcinogenesis. Fecal metabolites can be utilized, in addition to bacteria, for non-invasive diagnosis of colorectal neoplasia.

Video Abstract

**Supplementary Information:**

The online version contains supplementary material available at 10.1186/s40168-021-01208-5.

## Introduction

Colorectal cancer (CRC) remains a significant global health burden, with the gut microbiota identified as a key player in its development. Recent studies have shown that gut microbiota alteration can drive carcinogenesis by promoting hyperproliferation of colonic cells. During their colonization and propagation, gut bacteria produce an array of metabolites, which have both direct and indirect influence on host metabolism and immune responses. It has also been proposed that perturbation of the gut microbiota can enhance the production of carcinogenic products from damaging bacteria [1]. In particular, gut microbiota and their metabolites were shown to induce epigenetic modifications of host cells [2], with the metabolites acting as crucial messengers in the crosstalk [3]. *Fusobacterium nucleatum* is a commonly reported CRC-enriched microbe that increases gene methylation and induces microsatellite instability [4, 5]. Trimethylamine, mainly produced by *Escherichia coli,* induces DNA methylation [6] that is associated with CRC [7]. *Bilophila wadsworthia* and *Pyramidobacter spp* are other examples of CRC-enriched microbes, which reportedly enhanced carcinogenesis by producing genotoxic hydrogen sulphide in the gut [8–11]. On the other hand, certain gut bacteria such as *Faecalibacterium, Roseburia, Bifidobacterium*, *Eubacterium* and *Lactobacillus*, can ferment dietary fibers to short-chain fatty acids (SCFA), which are gut-protective and negatively associated with CRC. SCFAs including butyrate, propionate and acetate protect against CRC through mechanisms such as regulation of gut inflammation and immune system [12–14]. Butyrate and acetate can also act as inhibitors of histone deacetylase, thereby affecting the epigenetic modifications controlling CRC development [15].

The importance of microbiota-associated metabolites in colorectal carcinogenesis highlights the need to investigate the gut metabolome along the adenoma-carcinoma sequence to determine their mechanistic implications in CRC pathogenesis. To date, only few studies have simultaneously performed gut metagenomics and metabolomics from same subjects in order to resolve the interplay between gut microbiota and metabolites in colorectal tumorigenesis [16–19]. It is still not clear how and which microbes and metabolites interactively promote early events of CRC development.

Here, we integrated the gut metabolome and microbiota profiles of patients with CRC and colorectal adenomas (CRA) and compared them with those from healthy subjects. Our metabolite pathway enrichment and integrative analysis show that the gut metabolites and their association with gut microbiota were perturbed along colorectal carcinogenesis and that fecal metabolites can be utilized, in addition to bacteria, for non-invasive diagnosis of both CRA and CRC.

## Materials and methods

### Subjects and specimen collection

All 386 subjects underwent standard colonoscopy examinations at Prince of Wales Hospital, the Chinese University of Hong Kong, including 118 patients with CRC, 140 patients with CRA and 128 normal control participants. The average age of NC group was 64.03 years, 65.84 years for CRA group and 73.21 years for CRC group (Table S1). The distribution of gender and obesity among NC, CRA and CRC groups are shown in Table S1. All CRA and CRC subjects had intact colonic lesions at the time of stool collection. Stool samples were collected and stored at − 20 °C within 4 h and at − 80 °C within 24 h for long-term storage. Qiagen QIAmp DNA Stool Mini Kit (Qiagen) was used for DNA extraction according to the manufacturers’ instructions. All patients provided written informed consent for participation in this study. The study protocol was approved by the Clinical Research Ethics Committee of the Chinese University of Hong Kong.

### Metabolomics profiling

In order to identify metabolites that might be playing active roles in the relationship among gut microbiota, metabolites and CRC, we targeted a panel of metabolites that were previously implicated in human gut microbiota−host co-metabolism [20]. All samples were provided for gas chromatography coupled to time-of-flight mass spectrometer (GC-TOFMS) analysis using MicrobioMET (Metabo-Profile, Shanghai, P. R. China), based on automated alkyl chloroformate derivatization. The GC-TOFMS system (Pegasus HT, Leco Corp., St. Joseph, MO) was operated in electron ionization (EI) mode and was used to quantify the microbial metabolites. The raw data generated by GC-TOFMS were processed using XploreMET v2.0, (a proprietary software by Metabo-Profile, Shanghai, P. R China) for automatic baseline denoising, smoothing, peak picking, and peak signal alignment. The baseline offset was set to one. Five points were averaged for peak smoothing. Compound identification was implemented by comparing both retention time and MS similarity with reference standards. Details of sample preparation, reference standards, instrumentation and metabolites profiling are provided in Supplementary methods.

### Metabolomics data analysis

The metabolomics data analysis was conducted with R and online versions of MetaboAnalyst (http://www.metaboanalyst.ca) [21]. Partial least square discriminant analysis (PLS-DA) and principal component analysis (PCA) were performed using the R package mixOmics [22]. *P* values in both PCA and PLS-DA plot were calculated by permutational multivariate analysis of variance (PERMANOVA) using distance matrices through the R package vegan [23]. Differential metabolites analysis were conducted using the R package MetaboAnalystR [24]. The significantly altered metabolites were determined by variable importance in projection (VIP) scores from pairwise PLS-DA analysis and pairwise comparisons using the Wilcoxon rank-sum test. Benjamini-Hochberg false-discovery rate [25] (FDR) was used to correct for multiple comparison. Metabolites with VIP score > 1 and *p* values < 0.05 were considered significant. Interactions among disease associated metabolites were estimated by Spearman’s rank correlation. Metabolite set enrichment analysis (MSEA) was performed using the online tool MetaboAnalyst. All heatmaps were drawn using the R package Complex Heatmap [26] The workflow for the metabolomics analysis is shown in Fig. S1.

### Metagenomic sequencing and analysis

Whole-genome shotgun sequencing of all samples was carried out on an Illumina HiSeq 2000 (Illumina, San Diego, CA) platform. Trimmomatic v_0.36 was used to remove low quality sequences. Human sequences were removed after alignment with a reference genome (hg38 database) using Bowtie2 v_2.2.9, with default settings. Bacteria taxonomic profiles were obtained using MetaPhlAn 2.0 [27]. The average bacterial species level read count per sample was 2,316,872 ± 267,563. To reduce the effects of uneven sampling, the counts were rarefied to 1,947,705, the minimum read count of all samples. Bacterial taxa with < 20% prevalence were filtered out prior to downstream total sum scaling, differential abundance and biomarker selection analysis. Non-metric multidimensional scaling (NMDS) analysis was performed on Bray-Curtis distance from bacterial species abundances using the vegan R package. Differentially abundant bacterial species were identified by Kruskal-Wallis and Wilcoxon rank-sum tests. Benjamini-Hochberg false-discovery rate [25] (FDR) was used to correct for multiple comparison and adjusted *p*-values < 0.05 as the cut-off. The workflow for the metagenomics analysis is shown in Fig. S2.

### Integrative analyses of metabolomics profiling and metagenomics sequencing

Zero-inflated negative binomial (ZINB) regression (R package pscl), developed for modeling over-dispersed count outcome variables with excessive zeros, as found in microbial read counts data, was used to estimate the associations among metabolites and bacterial species. The read counts of bacterial species were treated as dependent variables in the ZINB regressions, while the concentrations of metabolites were considered as independent variables. The strengths of associations were measured by -log10(*p*-value)*sign (Beta) from the results of ZINB regressions, where Beta is the regression of the metabolite.

### Biomarker identification

Concentrations of metabolites were used to build classification models for metabolomic data while relative abundances of bacterial species were used as the inputs of classification models. Stepwise logistic regression models were built to discriminate paired groups using the function “glm” of R package stats. Biomarkers identification was performed by stepwise selection algorithm using the package MASS [28] in R. First, all significantly altered metabolites or bacterial species were included into the models as potential biomarkers. Then final biomarkers were identified by a stepwise model selection algorithm based on Akaike Information Criteria (AIC), which was performed using the R function “stepAIC” from package MASS. All identified biomarkers were then verified by random forest with 10-fold cross validation using the R package caret [29]. The receiver operating characteristic (ROC) analysis was conducted to illustrate performances of classification models, using R package pROC [30].

### Statistical analyses

All pairwise comparisons were performed using a two-sided Wilcoxon rank-sum test (Mann-Whitney U test). Multiple group comparisons were conducted using Kruskal-Wallis test. Fisher’s exact test was performed on categorical variables. The dissimilarity tests among groups (PERMANOVA) were conducted on Euclidean distance for metabolites and Bray-Curtis distance for bacteria, with 10,000 permutations in the R package, vegan. All statistical analyses were performed using R version 3.6.1.

## Results

### Alterations of gut metabolites in stages of CRC

**Fig. 1:**
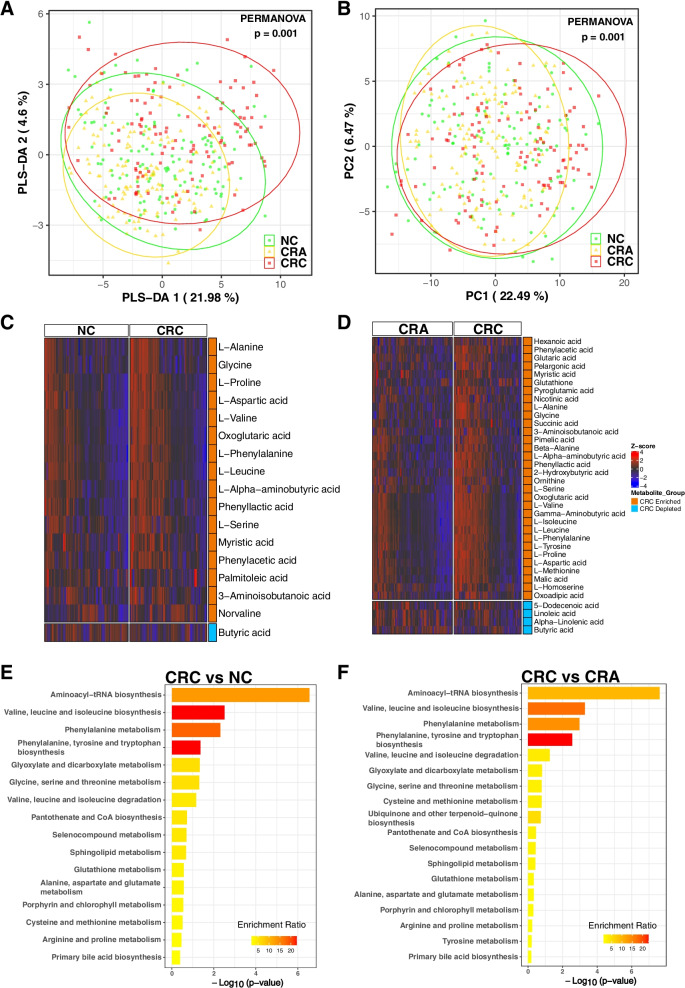
Metabolomic data profiles and pathway enrichment analysis. **A** Principal component analysis (PCA) for CRC, CRA and NC groups. **B** Partial least squares-discriminant analysis (PLS-DA) for CRC, CRA and NC groups. **C** Z-score heatmap of 17 significantly altered metabolites between CRC and NC. **D** Z-score heatmap of 36 significantly altered metabolites between CRC and CRA. Significantly altered metabolites were determined using VIP score from pairwise PLD-DA analysis and Wilcoxon rank-sum test, with VIP > 1 and *p* < 0.05 as the cut-off for significance. CRC; colorectal cancer, CRA; colorectal adenoma, NC; normal control. **E** Metabolomic pathway enrichment analysis using the 17 significantly altered metabolites between CRC and NC. **F** Metabolomic pathway enrichment analysis using the 36 significantly altered metabolites between CRC and CRA. CRC; colorectal cancer, CRA; colorectal adenoma, NC; normal control

Our study included 386 subjects, namely 118 patients with CRC, 140 patients with CRA and 128 healthy subjects as normal control (NC). A total of 97 metabolites were quantified from stool samples using GC-TOFMS. PLS-DA (Fig. 1A) and PCA (Fig. 1B) showed that there are differences in the gut metabolite profiles among CRC, CRA and NC groups (PERMANOVA, both *p* = 0.001), indicating a gut-metabolite shift in colon carcinogenesis.

To identify significantly altered metabolites that may be important across the stages of CRC development, we performed pairwise comparisons between groups. When NC was compared with CRC, 17 metabolites were significantly altered. These include the enrichment of L-alanine, glycine, L-proline, L-aspartic acid, L-valine, L-leucine, L-serine, myristic acid, phenyl lactic acid, oxoglutaric acid, L-phenylalanine, L-alpha-aminobutyric acid, phenylacetic acid, palmitoleic acid, 3-aminoisobutanoic acid and norvaline. In contrast, butyric acid was depleted in CRC patients compared with NC (Fig. 1C and Fig. S3A, Table S2). With the comparison of CRA with CRC, 36 metabolites were differentially abundant, including the depletion of 5-dodecenoic acid, linoleic acid, alpha-linolenic acid and butyric acid in CRC (Fig. 1D and Fig. S3B, Table S3). Interestingly, L-alanine, glycine, L-proline, L-aspartic acid, L-valine, L-leucine, L-serine, myristic acid and phenyl lactic acid were enriched in CRC compared to both NC and CRA subjects (Fig. 1C and D, Fig. S3). Moreover, norvaline and myristic acid were found to show increasing trends from NC, through CRA, to CRC (Fig. S4), suggesting their potential contribution to the progression of colon tumorigenesis.

To gain insight into the functions of significantly altered metabolites for each paired group, we conducted MSEA. We observed differences in pathways associated with the metabolism of branched-chain amino acids (BCAAs) in stages leading to CRC. The top 4 enriched pathways in CRC compared with NC (Fig. 1E) and CRA (Fig. 1F) were (1) aminoacyl-tRNA biosynthesis, (2) valine, leucine and isoleucine biosynthesis, (3) phenylalanine metabolism and (4) phenylalanine, tyrosine and tryptophan biosynthesis, indicating that metabolic pathways are altered in addition to individual metabolites in colorectal carcinogenesis.

### Metabolites as CRC diagnostic markers

**Fig. 2:**
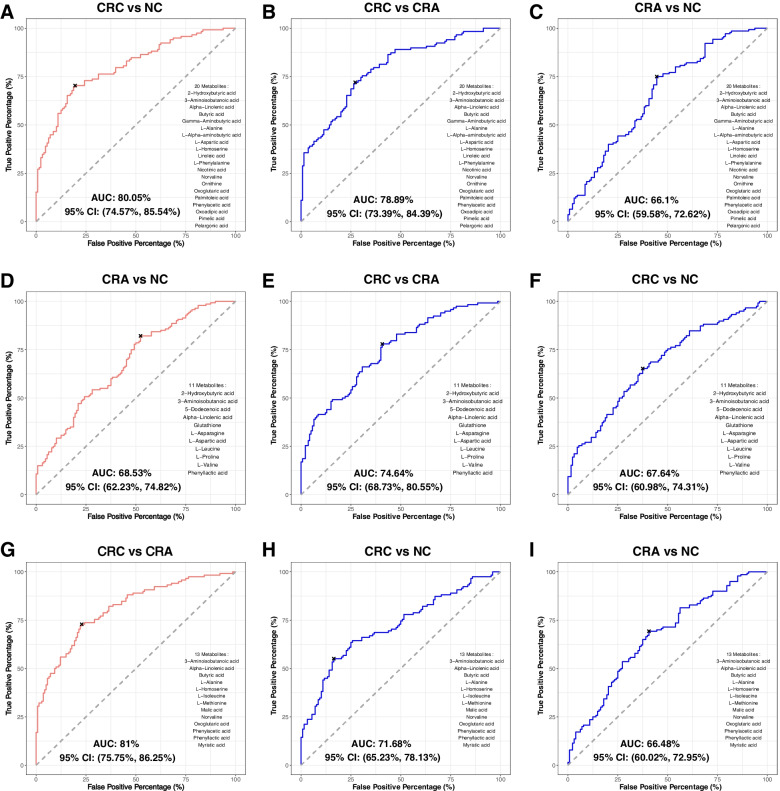
Metabolite markers for pairwise discriminations of CRC, CRA and NC groups. **A** Receiver operating characteristic (ROC) analysis for the 20 metabolite markers discriminating CRC from NC. **B** ROC analysis applying the 20 CRC vs NC metabolite markers to discriminate CRA from NC. **C** ROC analysis applying the 20 CRC vs NC metabolite markers to discriminate CRC from CRA. **D** ROC analysis for the 11 metabolite markers discriminating CRA from NC. **E** ROC analysis applying the 11 CRA vs NC metabolite markers to discriminate CRC from NC. **F** ROC analysis applying the 11 CRA vs NC metabolite markers to discriminate CRC from CRA. **G** ROC analysis for the 13 metabolite markers discriminating CRC from CRA. **H** ROC analysis applying the 13 CRC vs CRA metabolite markers to discriminate CRC from NC. **I** ROC analysis for applying the 13 CRC vs CRA metabolite markers to discriminate CRA from NC. CRC; colorectal cancer, CRA; colorectal adenoma, NC; normal control

We further explored the potential use of gut microbiome-associated metabolites for non-invasive diagnosis of CRC. Using the identified significantly altered metabolites (Fig. 1C and D), we built stepwise logistic regression models for the classifications of paired groups. Our model selected 20 metabolites as markers to classify CRC from NC subjects, with an area under the curve (AUC) of 0.80 (Fig. 2A). The same 20 markers distinguished CRC from CRA with an AUC of 0.7889 (Fig. 2B), and CRA from NC with an AUC of 0.661 (Fig. 2C). To discriminate CRA from NC, 11 metabolites markers were identified with an AUC of 0.6853 (Fig. 2D). These 11 metabolites markers classified CRA from CRC with an AUC of 0.7464 (Fig. 2E), and CRC from NC with an AUC of 0.6764 (Fig. 2F). CRC was classified from CRA by 13 metabolites markers with an AUC of 0.81 (Fig. 2G). With these 13 markers, AUCs of 0.7168 and 0.6648 were obtained for CRC vs NC and CRA vs NC, respectively (Fig. 2H and I). Moreover, adjusting clinical features, namely age, gender and obesity improved the performance of all markers with increases of about 8% in the AUCs (Fig. S5). The performances of the identified markers were validated by random forest with 10-fold cross validation (Fig. S6).

### Bacterial species as CRC diagnostic markers

**Fig. 3:**
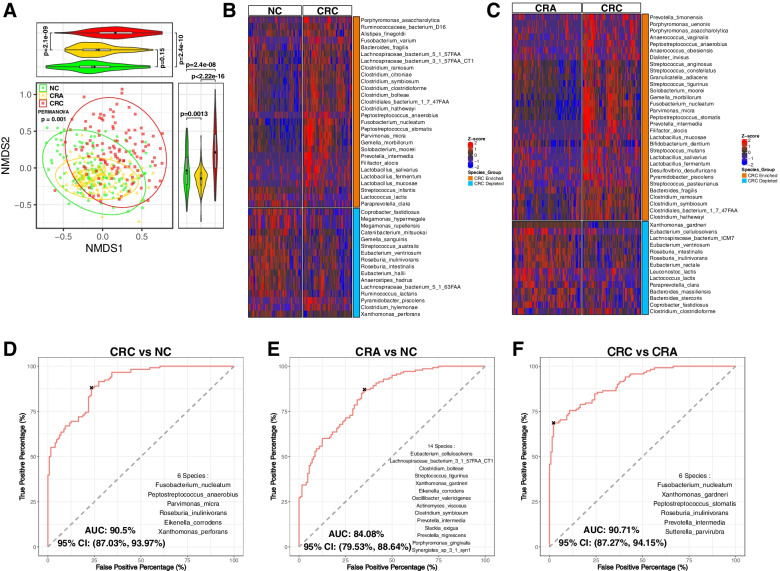
Metagenomic data profiles and diagnostic performances. **A** Non-metric multidimensional scaling (NMDS) analysis on the Bray-Curtis distance from bacterial species abundances for CRC, CRA and NC groups. **B** Heatmap of 44 differentially abundant bacterial species between CRC and NC. **C** Heatmap of 45 differentially abundant bacterial species between CRC and CRA. Differentially abundant species were determined using Wilcoxon rank-sum test for the relative abundance data, with cut-off: FDR adjusted *p* < 0.05, mean of relative abundance > 0.001 and prevalence rate > 0.4. CRC; colorectal cancer, CRA; colorectal adenoma, NC; normal control. **D** Receiver operating characteristic (ROC) analysis for the 6 bacteria discriminating CRC from NC. **E** ROC analysis for the 14 bacteria markers discriminating CRA from NC. **F** ROC analysis for the 6 bacteria markers discriminating CRC from CRA. CRC; colorectal cancer, CRA; colorectal adenoma, NC; normal control

Bacterial dysbiosis is associated with colon tumorigenesis [31]. We further investigated the differential distribution of bacteria along CRC stages using fecal shotgun metagenomics sequences from all subjects. Analysis of beta diversity via NMDS revealed bacterial communities to differ among CRC, CRA and NC groups (*p* = 0.001; Fig. 3A). Several bacterial species, including *Peptostreptococcus stomatis, Fusobacterium nucleatum, Parvimonas micra, Peptostreptococcus anaerobius* and *Bacteroides fragilis*, were enriched in CRC compared to NC (Fig. 3B) and subjects with CRA (Fig. 3C) while others such as *Coprobacter fastidosus, Eubacterium ventriosum, Roseburia interinalis and Roseburia inulivorans* were depleted in CRC patients compared to NC (Fig. 3B) and subjects with CRA (Fig. 3C). *Leptotrichia buccalis* and *Prevotella veroralis* increased (Fig. S7A) while *Lachnospiraceae bacterium 1_4_56FAA* and *Eubacterium dolichum* decreased (Fig. S7B) sequentially from NC, through CRA, to CRC.

Furthermore, we used stepwise logistic regression models to identify potential diagnostic bacterial species. *F. nucleatum*, *P. anaerobius*, *P. micra*, *R. inulinivorans, E. corrodens* and *X. perforans* classified CRC from NC with an AUC of 0.905, consistent with our previous study [32] (Fig. 3D). These 6 bacterial markers separated CRC from CRA with an AUC of 0.8877 (Fig. S8A), and CRA from NC with an AUC of 0.602 (Fig. S8B). CRA and NC were discriminated by 14 bacterial species with an AUC of 0.8408 (Fig. 3E). The 14 markers also classified CRC from NC with an AUC of 0.8207 (Fig. S8C) and CRC from CRA with an AUC of 0.8925 (Fig. S8D). CRC was classified from CRA with an AUC of 0.9071 by 6 bacterial markers including *F. nucleatum* (Fig. 3F). The 6 markers discriminated CRC from NC, and CRA from NC, with an AUC of 0.8545 and 0.7188 respectively (Fig. S8E and S8F). We further verified the bacterial markers by random forest with 10-fold cross validation. Compared with the metabolite markers, AUCs achieved by the bacterial markers were not improved by adjusting for age, gender and obesity (Fig. S9 and Fig. S10).

### Bacterial markers improve diagnostic performance of metabolites markers

**Fig. 4:**
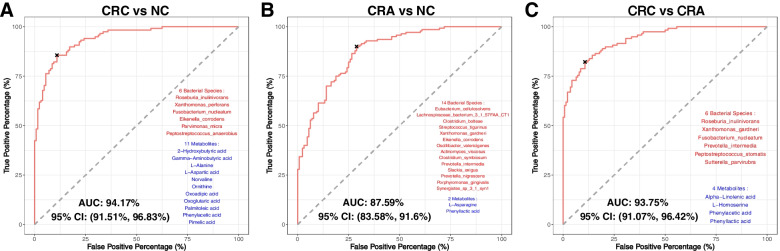
Combination of bacteria and metabolite markers for pairwise discriminations and their interactions in CRC, CRA and NC groups. **A** Receiver operating characteristic (ROC) analysis for the combination of 6 bacteria and 11 metabolites discriminating CRC from NC. **B** ROC analysis for the combination of 14 bacteria and 2 metabolite markers discriminating CRA from NC. **C** ROC analysis for the combination of 6 bacterial and 4 metabolite markers discriminating CRC from CRA. CRC; colorectal cancer, CRA; colorectal adenoma, NC; normal control

To investigate whether better discrimination among the stages of colorectal carcinogenesis can be achieved, we combined metabolite and bacterial markers using stepwise logistic regression. For classifying CRC from NC, 11 metabolite markers (2-hydroxybutyric acid, gamma-aminobutyric acid, L-alanine, L-aspartic acid, norvaline, ornithine, oxoadipic acid, oxoglutaric acid, palmitoleic acid, phenylacetic acid and pimelic acid) and 6 bacterial species (*F. nucleatum*, *P. anaerobius*, *P. micra*, *R. inulinivorans, E. corrodens* and *X. perforin*) achieved a higher AUC of 0.9417 (Fig. 4A), compared with an AUC of 0.905 with only metabolite markers. The combined metabolite and bacterial markers also discriminated CRA from NC with an AUC of 0.6728 (Fig. S11A) and CRC from CRA with an AUC of 0.92 (Fig. S11B). Inclusion of L-asparagine and phenyl lactic acid with 14 CRA-versus-NC bacterial markers improved the AUC from 0.8408 to 0.8759 (Fig. 4B). This combination classified CRC from NC with an AUC of 0.8195 (Fig. S11C) and CRC from CRA with an AUC of 0.8976 (Fig. S11D). Furthermore, the combination of alpha-linolenic acid, L-homoserine, phenylacetic acid and phenyl lactic acid with 6 bacterial markers increased AUC from 0.9071 to 0.9375 in classifying CRC from CRA (Fig. 4C). The combination of 10 metabolites and bacterial markers for distinguishing CRC and CRA also classified CRC from NC and CRA from NC, with AUCs of 0.8723 and 0.7499, respectively (Fig. S11E and S11F), demonstrating potential for early diagnosis of CRA.

### Interactions among metabolites and bacteria are altered in CRC

To understand the potential interplay between significantly altered metabolites and differentially abundant bacterial species, we performed association analysis using ZINB regression. The distribution of associations was significantly different across the CRC stages (Fig. S12). Moreover, some associations followed an increasing or decreasing trend along CRC progression while some were reversed from negative to positive associations (Fig. S13A) or vice versa (Fig. S13B). Among the 6 bacterial species discriminating CRC from NC, the association between *P. anaerobius* and glycine was significant and increased along CRC progression (*p* < 0.05, Table S4), while the association between *P. anaerobius* and myristic acid was significant but decreased in CRC. *P. micra* was significantly associated with linoleic acid and L-valine and both associations followed decreasing trend from NC, through CRA, to CRC. Among the 14 bacterial markers discriminating CRA from NC, *Clostridium symbiosum* was significantly correlated with CRC-enriched L-valine and L-homoserine and the strengths of both associations increased along CRC progression. Moreover, *Synergistes* sp. 3_1_syn1 was significantly associated with L-aspartic acid and L-tyrosine and both associations followed a decreasing trend. The correlations between *Porphyromonas gingivalis* and gamma-aminobutyric acid as well as between *Prevotella nigrescens* and L-asparagine were also significant and decreased along CRC progression (Table S4).

We further investigated the interactions among CRC-associated metabolites. The CRC-depleted metabolite butyric acid showed co-exclusive interactions with CRC-enriched metabolites including pimelic acid, L-proline, L-methionine, and L-isoleucine. Moreover, there were strong co-occurrence relationships (correlation coefficient > 0.6 and p < 0.05) among CRC-enriched metabolites, such as L-proline, L-aspartic acid, L-methionine, oxoglutaric acid, L-leucine, L-valine, gamma-aminobutyric acid, L-isoleucine, L-phenylalanine and L-tyrosine supporting their potential role in CRC (Fig. S14). Taken together, these results suggest that there are significant associations among gut metabolites and bacteria, which are changed along the stages of colorectal carcinogenesis.

## Discussion

Accumulating evidence reveals that the gut microbiota and their metabolites play important roles in colorectal tumorigenesis. Here, we profiled the fecal metabolites and microbiome of CRC patients and compared them with those of precancerous CRA patients and healthy subjects. We demonstrated that key metabolic pathways were disrupted along CRC pathogenesis. Integrated metabolomic and microbiome analysis showed that interactions among CRC associated bacteria and metabolites are altered along the development of CRC. Importantly, we demonstrate a promising potential of fecal metabolites, in addition to bacteria, for non-invasive diagnosis of CRC.

Metabolomics has the potential for diagnosis of cancer including CRC [16, 33]. Our metabolic profiling revealed that several amino acids, namely L-alanine, glycine, L-proline, L-aspartic acid, L-valine, L-leucine, L-serine, myristic acid and phenyl lactic acid were enriched in CRC patients compared to CRA and NC groups of subjects. Amino acids play important roles in several steps of molecular biosynthesis where they maintain redox balance and serve as energy sources [34]. Abundant amino acids have also been reported to be crucial in driving the proliferation of cancer cells [35]. Derivatives of amino acids can affect immune responses and regulate epigenetics. As such, they are reportedly associated with carcinogenesis [36]. Alanine, which was identified to be CRC-associated in this study, had been reported as an important survival signal in some gastrointestinal cancers. For example, stromal cells secrete alanine required by the TCA cycle in promoting pancreatic cancer growth [37]. Additionally, glycine can provide essential precursors for the synthesis of nucleic acids, lipids and proteins, which support growth of cancer cells [38]. It was also reported that proline biosynthesis was upregulated and associated with poor prognosis of cancer [39], supporting our findings in this study. Interestingly, norvaline and myristic acid were found to show increasing trend from NC to CRA, and to CRC. Norvaline is an isomer of valine, which was implicated in the cytotoxic activity of macrophages against breast tumor cells [40]. Norvaline also reportedly promotes tissue regeneration and muscle growth partially by the inhibition of ribosomal protein S6 kinase beta-1 [41]. Myristic acid is a common unsaturated fatty acid positively associated with high cholesterol levels in human and reported to increase the risk of breast cancer development [42]. The increasing trend of these two metabolites along CRC development hints at their potential roles in colorectal tumorigenesis and warrants further investigation. The only metabolite depleted in CRC patients in this study is butyric acid, a short chain fatty acid produced by fermentation of dietary fibers in the large bowel. It has been consistently demonstrated that butyric acid has a protective effect against colorectal cancer by inhibiting cell proliferation and inducing apoptosis [43], further supporting our discovery in this study.

Pathway enrichment analysis showed that aminoacyl-tRNA biosynthesis, aromatic amino acids biosynthesis and BCAAs metabolisms were altered in CRC patients and adenoma patients compared with healthy subjects. Aminoacyl-tRNA biosynthesis needs aminoacyl-tRNA synthetases, an important class of enzymes with an evolutionarily conserved mechanism for protein synthesis, some of which show positive associations with colorectal tumor development [44]. BCAAs including valine, leucine and isoleucine were CRC-upregulated in this study compared to CRA and healthy subjects. They are essential nutrients for cancer growth and are used by tumors in various biosynthetic pathways and as sources of energy [45]. Moreover, gut microbes were observed to play active roles in the metabolism of aromatic amino acids including tyrosine, phenylalanine and tryptophan [46]. Modulation of the serum level of aromatic amino acids was shown to impair both intestinal permeability and systemic immunity in gnotobiotic mice [46]. This suggests that dysregulation of aromatic amino acid biosynthesis observed in this study may induce CRC through an impaired gut barrier. Also, tryptophan metabolism was reportedly implicated in therapy against gastrointestinal disorders through the host-gut microbiota interface [47]. Phenylalanine, found upregulated in CRC patients in this study, is an essential amino acid, which may contribute to proliferation and migration of cancer cells [48]. Our discovery, supported by previous reports [16, 49] show that BCAAs, aromatic amino acids and phenylalanine metabolomic pathways may play important roles in colorectal carcinogenesis.

We further explored the potential use of gut microbiome associated metabolites in non-invasive diagnosis of CRC. CRC was classified from NC and from CRA with 20 and 13 metabolite markers, respectively, each with an AUC of about 0.80. Six bacterial species distinguished CRC from NC and CRA with an AUC of 0.91 and 0.89 respectively. With the combination of metabolites and bacterial markers, a higher discriminating power demonstrated by an AUC of 0.94 was achieved with 11 metabolites and 6 bacterial species including *F. nucleatum*, *P. anaerobius*, *P. micra*, *R. inulinivorans, E. corrodens* and *X. perforans.* Interestingly, we observed that a combination of 4 metabolites namely alpha linoleic acid, L-homoserine, phenyl lactic acid and phenyl acetic acid, and bacteria markers including *F. nucleatum*, *P. anaerobius*, *P. micra*, *R. inulinivorans, E. corrodens* and *X. perforans* classified CRA from NC, with an AUC of 0.7499 demonstrating the potential for early diagnosis of colorectal adenoma from healthy patients.

Moreover, our association analysis revealed that the relationships among metabolites and bacteria were significantly different in CRC patients compared with NC and CRA subjects. While some associations followed increasing or decreasing trends along CRC progression, some were reversed from negative to positive and vice versa. Notable is the increased correlations between glycine, reported to support cancer cell growth [38], and *P. anaerobius* that drives CRC via the PI3K-Akt-NF-κB signaling pathway [49], suggesting a potential cooperation between glycine and *P. anaerobius* in the development of CRC. In addition, *Clostridium symbiosum,* which was found to increase in abundance from the colon tissues of healthy subjects to adenoma patients and finally to colonic cancer patients [50], was significantly correlated with CRC-enriched metabolites L-valine and L-homoserine, with increased strengths along CRC progression. Collectively, these results indicate significant interplays among gut metabolites and bacteria, which might influence colorectal carcinogenesis.

The microbial related metabolites reported in this study were based on metabolomics data and chemical properties of human microbiome associated metabolites [20, 51]. Automated alkyl chloroformate derivatization method was used for the GC-TOF/MS detection [20]. The GC-MS response was poor for bile acids due to their strong polarity. New methods have recently been developed for the specific detection of bile acids [52], and its association with CRC will be examined in the future study.

In conclusion, our integrated metabolites and microbiome study demonstrates that gut metabolites along with the microbiome are altered along stages of colorectal carcinogenesis and that the combination of metabolites and bacterial taxa can increase the chance of non-invasive diagnosis of colorectal cancer and adenoma. This study underscores potential early-driver metabolites in CRC tumorigenesis and informs further experiments towards the development of better CRC diagnosis and prevention strategies.

## Supplementary Information

**Additional file 1: Table S1.** Demographic and clinical details of samples.

**Additional file 2: Table S2.** Differential test results for CRC vs NC.

**Additional file 3: Table S3**. Differential test results for CRC vs CRA.

**Additional file 4: Table S4.** The list of significant associations among metabolites and bacteria following direct trends.

**Additional file 5.** Supplementary Methods.

**Additional file 6: Figure S1.** The workflow for metabolomics data analysis.

**Additional file 7: Figure S2.** The workflow for metagenomics data analysis.

**Additional file 8: Figure S3.** Volcano plots of significantly altered metabolites between groups.

**Additional file 9: Figure S4.** Significantly altered metabolites show direct trends along CRC progression. Pairwise comparisons were performed using Wilcoxon rank-sum test. CRC; colorectal cancer, CRA; colorectal adenoma, NC; normal control.

**Additional file 10: Figure S5.** Metabolites markers for pairwise discriminations of CRC, CRA and NC groups with adjustment of age, gender and obesity.

**Additional file 11: Figure S6.** Validation of metabolites markers for pairwise discriminations of CRC, CRA and NC groups by random forest model with 10-fold cross validation.

**Additional file 12: Figure S7.** Differentially abundant bacterial species show direct trends along CRC progression.

**Additional file 13: Figure S8.** Bacterial species markers for pairwise discriminations of CRC, CRA and NC groups.

**Additional file 14: Figure S9.** Bacterial species markers for pairwise discriminations of CRC, CRA and NC groups with adjustment of age, gender and obesity.

**Additional file 15: Figure S10.** Validation of bacterial species markers for pairwise discriminations of t CRC, CRA and NC groups by random forest model with 10-fold cross validation.

**Additional file 16: Figure S11.** Combination of bacteria and metabolites markers for pairwise discriminations of CRC, CRA and NC groups.

**Additional file 17: Figure S12.** Distributions of association between significantly altered metabolites and bacterial species for CRC, CRA and NC groups.

**Additional file 18: Figure S13.** Interactions among metabolites and bacteria are altered in CRC.

**Additional file 19: Figure S14.** Heatmap of correlations between disease associated metabolites. The correlation strengths were measured by Spearman’s rank correlation coefficient. Only correlation coefficients with *p* > 0.05 were shown on the heatmap. The size of the circles are proportional to the correlation strength.

## Data Availability

The data and materials that support the findings in this study are available from the corresponding author, Prof. Jun Yu, upon reasonable request.

## Abbreviations

AIC: Akaike Information Criteria
AUC: Area under the curve
BCAAs: Branched-chain amino acids
CRC: Colorectal cancer
CRA: Colorectal adenomas
EI: Electron ionization
FDR: False-discovery rate
GC-TOFMS: Gas chromatography coupled to time-of-flight mass spectrometer
MSEA: Metabolite set enrichment analysis
NC: Normal controls
NMDS: Non-metric multidimensional scaling
PCA: Principal component analysis
PERMANOVA: Permutational multivariate analysis of variance
PLS-DA: Partial least square discriminant analysis
ROC: Receiver operating characteristic
SCFA: Short-chain fatty acids
ZINB: Zero-inflated negative binomial
