# Supplementary material for: Altered gut metabolites and microbiota interactions are implicated in colorectal carcinogenesis and can be non-invasive diagnostic biomarkers
Source: Microbiome. 2022 Feb 21;10:35. doi: 10.1186/s40168-021-01208-5 (PMC8862353; doi:10.1186/s40168-021-01208-5)
Supplement: Supplementary file 6 — Additional file 5. Supplementary Methods. [file 40168_2021_1208_MOESM6_ESM.docx]

**Altered gut metabolites and microbiota interactions are implicated in** **colorectal carcinogenesis and can be non-invasive diagnostic biomarkers**

**Supplementary Methods**

**Metabolomics Profiling**

The study was designed to assess intestinal microbial metabolite profiles in samples using MicrobioMET (Metabo-Profile, Shanghai, P.R. China) [1], comprising a panel of abundant microbial metabolites including aromatic phenols and indoles, phenolic acids, short-chain fatty acids and branched-chain amino acids, amino acids, and organic acids. The reference library was developed consists of 132 methyl and ethyl chloroformate (MCF and ECF) derivatized compounds. A list of Reference Chemicals is provided in the reference paper [1]. Quantification was done using gas chromatography coupled to time-of-flight mass spectrometer (GC-TOFMS).

**Sample Preparation**

A total of 386 samples were submitted to the Metabo-Profile and immediately stored at -80°C freezer. The sample preparation and derivatization protocols were based on previously published procedures with modifications [1,2]. Samples were thawed on ice-bath to diminish sample degradation. Approximately 50 mg of the samples was homogenized with 300 μL of NaOH (1M) solution using a homogenizer (BB24, Next Advance, Inc., Averill Park, NY, USA) and centrifuged at 13, 500 rpm and 4 °C for 20 min (Microfuge 20R，Beckman Coulter, Inc., Indianapolis, IN, USA). This was followed by transfer of 200 μL of supernatant into an autosampler vial (Agilent Technologies, Foster City, CA, USA). The residue was further extracted with 200 μL of cold methanol. After the second step of homogenization and centrifugation, 167 μL each of supernatant was combined with the first supernatant in the autosampler vial.

The extracts in the autosampler vial were submitted for automated sample derivatization with a robotic multipurpose sample MPS2 with dual heads (Gerstel, Muehlheim, Germany). Briefly, each 20 μL of MCF was added to the mixture and the sample was vortexed vigorously for 30 s. Another 20 μL of MCF was added for the second round of derivatization. Four hundred microliter of chloroform followed by four hundred microliter of sodium bicarbonate solution (50 mM) was added to achieve separation. The prepared samples were centrifuged at 4°C and 4000 g for 20 min, and the bottom chloroform layer was carefully transferred by robotic preparation station to a capped empty autosampler vial preloaded with approximately 25 mg of anhydrous sodium sulfate. The sample pretreated with sodium sulfate was shaken on a laboratory shaker at 1,500 rpm and 4°C for 20 min and further transferred to a capped empty autosampler vial for injection.

**Instrumentation**

A gas chromatography coupled to time-of-flight mass spectrometry (GC-TOFMS) system (Pegasus HT, Leco Corp., St. Joseph, MO, USA) operated in electron ionization (EI) mode was used to quantitate the microbial metabolite. The optimized instrument settings are briefly described below. Instrument optimization was performed every 24 hours.


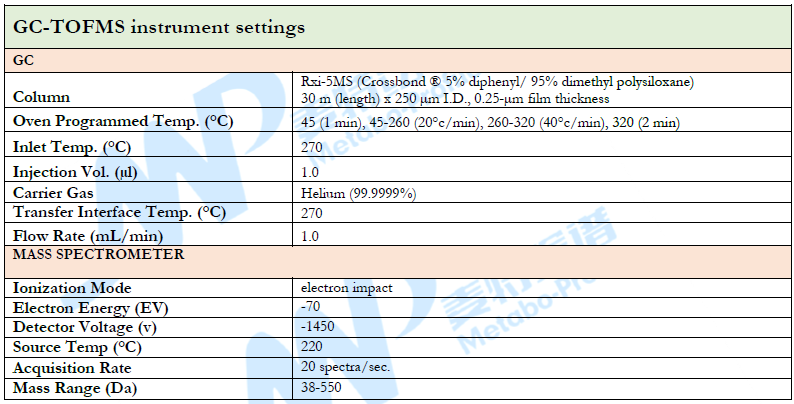


The raw data generated by GC-TOFMS was processed using XploreMET v2.0, (a proprietary software by Metabo-Profile, Shanghai, P.R China) for automatic baseline denoising, smoothing, peak picking, and peak signal alignment. The baseline offset was set to one. Five points were averaged for peak smoothing. Compound identification was performed by comparing both retention time and MS similarity with reference standards. [1].

**Chemicals and Reagents**

The stock solutions of all 132 representative reference chemicals of microbial metabolites were prepared in methanol, ultrapure water, or sodium hydroxide solution at a concentration of either 5 mg/mL or 1 mg/mL. The three groups of stock solutions were mixed to create stock calibration solutions based on their solubility and chemical properties. Further serial dilutions of the stock calibrations were made to obtain eight calibrators at a wide concentration ranging from 0.008 to 250 μg/mL. Internal standards were added to monitor the data quality and compensate for matrix effects.

**References**

1. Zhao L, Ni Y, Su M, Li H, Dong F, Chen W, et al. High Throughput and Quantitative Measurement of Microbial Metabolome by Gas Chromatography/Mass Spectrometry Using Automated Alkyl Chloroformate Derivatization. Anal Chem. 2017;89:5565–77.

2. Martin F-P, Su M-M, Xie G-X, Guiraud SP, Kussmann M, Godin J-P, et al. Urinary metabolic insights into host-gut microbial interactions in healthy and IBD children. WJG. 2017;23:3643.
