# Supplementary material for: Altered gut metabolites and microbiota interactions are implicated in colorectal carcinogenesis and can be non-invasive diagnostic biomarkers
Source: Microbiome. 2022 Feb 21;10:35. doi: 10.1186/s40168-021-01208-5 (PMC8862353; doi:10.1186/s40168-021-01208-5)
Supplement: Supplementary file 7 — Additional file 6: Figure S1. The workflow for metabolomics data analysis. [file 40168_2021_1208_MOESM7_ESM.pptx]

## Slide 1
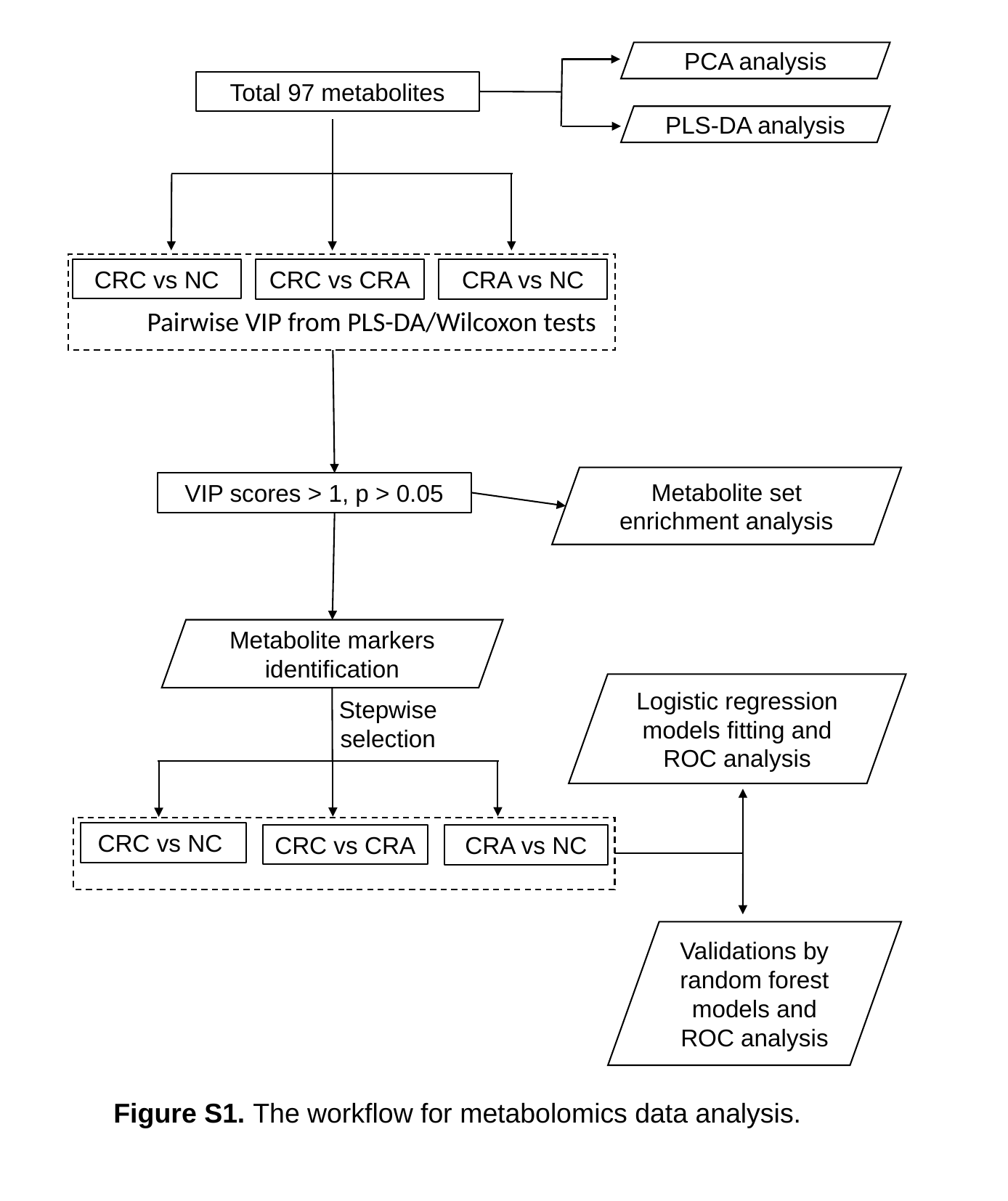

PCA analysis
Total 97 metabolites
PLS-DA analysis
CRC vs NC
CRC vs CRA
CRA vs NC
Pairwise VIP from PLS-DA/Wilcoxon tests
Metabolite set enrichment analysis
VIP scores > 1, p > 0.05
Metabolite markers identification
Logistic regression models fitting and ROC analysis
Stepwise selection
CRC vs NC
CRC vs CRA
CRA vs NC
Validations by random forest models and ROC analysis
Figure S1. The workflow for metabolomics data analysis.
