# Supplementary material for: Altered gut metabolites and microbiota interactions are implicated in colorectal carcinogenesis and can be non-invasive diagnostic biomarkers
Source: Microbiome. 2022 Feb 21;10:35. doi: 10.1186/s40168-021-01208-5 (PMC8862353; doi:10.1186/s40168-021-01208-5)
Supplement: Supplementary file 8 — Additional file 7: Figure S2. The workflow for metagenomics data analysis. [file 40168_2021_1208_MOESM8_ESM.pptx]

## Slide 1
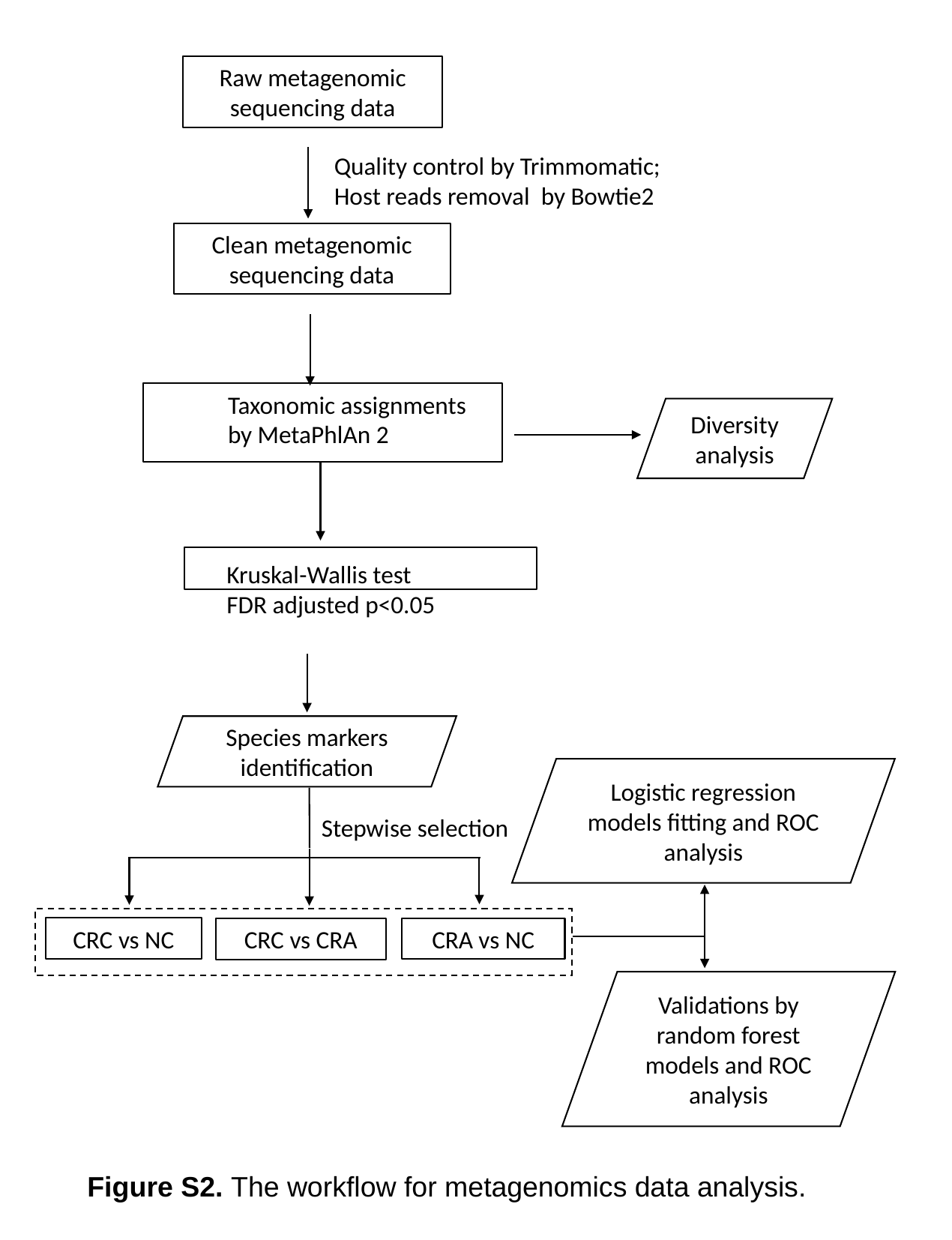

Raw metagenomic sequencing data
Quality control by Trimmomatic;
Host reads removal by Bowtie2
Clean metagenomic sequencing data
Taxonomic assignments
by MetaPhlAn 2
Diversity analysis
Kruskal-Wallis test
FDR adjusted p<0.05
Species markers identification
Logistic regression models fitting and ROC analysis
Stepwise selection
CRC vs NC
CRA vs NC
CRC vs CRA
Validations by random forest models and ROC analysis
Figure S2. The workflow for metagenomics data analysis.
