# Supplementary material for: Altered gut metabolites and microbiota interactions are implicated in colorectal carcinogenesis and can be non-invasive diagnostic biomarkers
Source: Microbiome. 2022 Feb 21;10:35. doi: 10.1186/s40168-021-01208-5 (PMC8862353; doi:10.1186/s40168-021-01208-5)
Supplement: Supplementary file 9 — Additional file 8: Figure S3. Volcano plots of significantly altered metabolites between groups. [file 40168_2021_1208_MOESM9_ESM.pptx]

## Slide 1
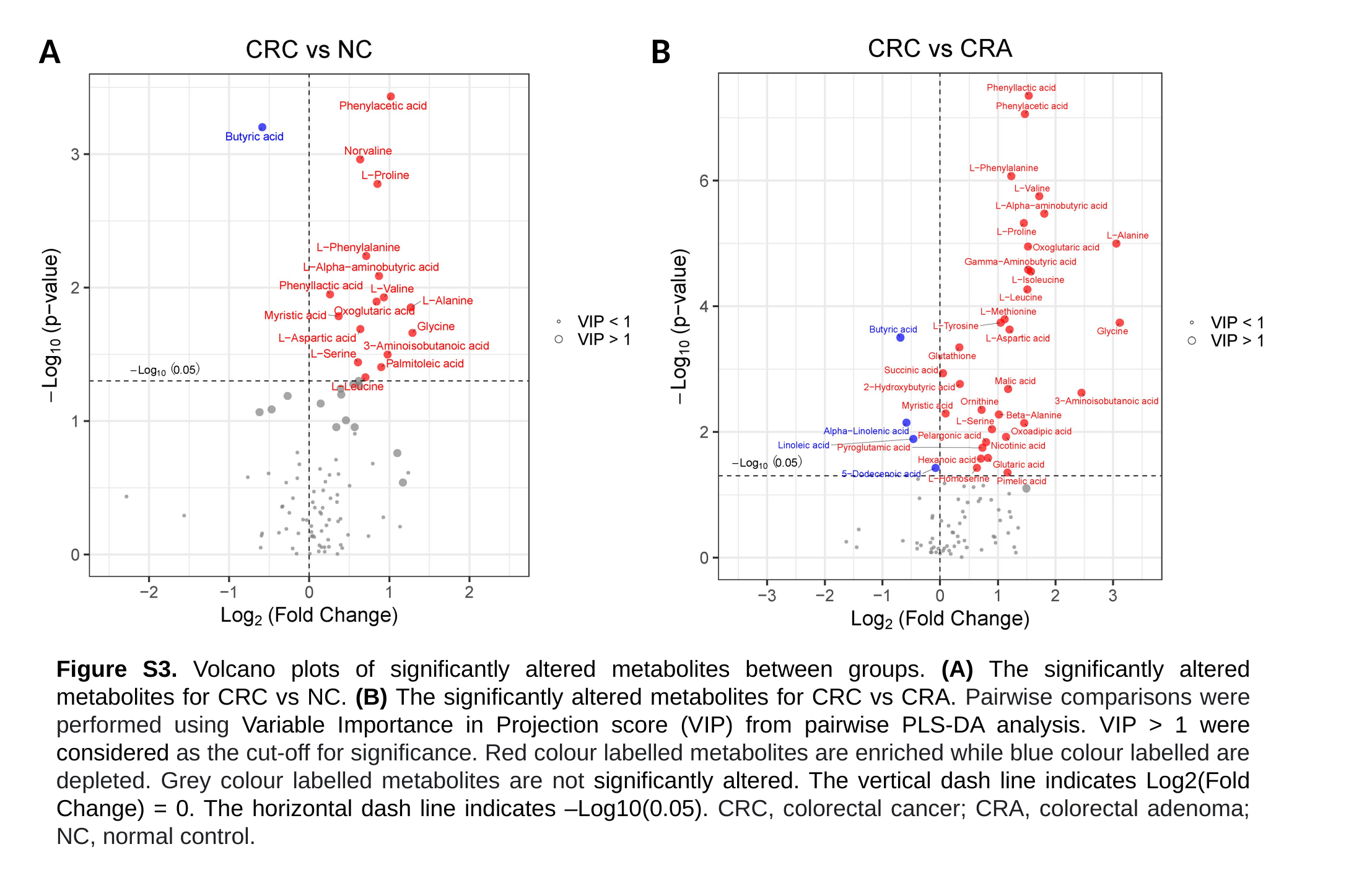

A
B
Figure S3. Volcano plots of significantly altered metabolites between groups. (A) The significantly altered metabolites for CRC vs NC. (B) The significantly altered metabolites for CRC vs CRA. Pairwise comparisons were performed using Variable Importance in Projection score (VIP) from pairwise PLS-DA analysis. VIP > 1 were considered as the cut-off for significance. Red colour labelled metabolites are enriched while blue colour labelled are depleted. Grey colour labelled metabolites are not significantly altered. The vertical dash line indicates Log2(Fold Change) = 0. The horizontal dash line indicates –Log10(0.05). CRC, colorectal cancer; CRA, colorectal adenoma; NC, normal control.
