# Supplementary material for: Altered gut metabolites and microbiota interactions are implicated in colorectal carcinogenesis and can be non-invasive diagnostic biomarkers
Source: Microbiome. 2022 Feb 21;10:35. doi: 10.1186/s40168-021-01208-5 (PMC8862353; doi:10.1186/s40168-021-01208-5)
Supplement: Supplementary file 10 — Additional file 9: Figure S4. Significantly altered metabolites show direct trends along CRC progression. Pairwise comparisons were performed using Wilcoxon rank-sum test. CRC; colorectal cancer, CRA; colorectal adenoma, NC; normal control. [file 40168_2021_1208_MOESM10_ESM.pptx]

## Slide 1
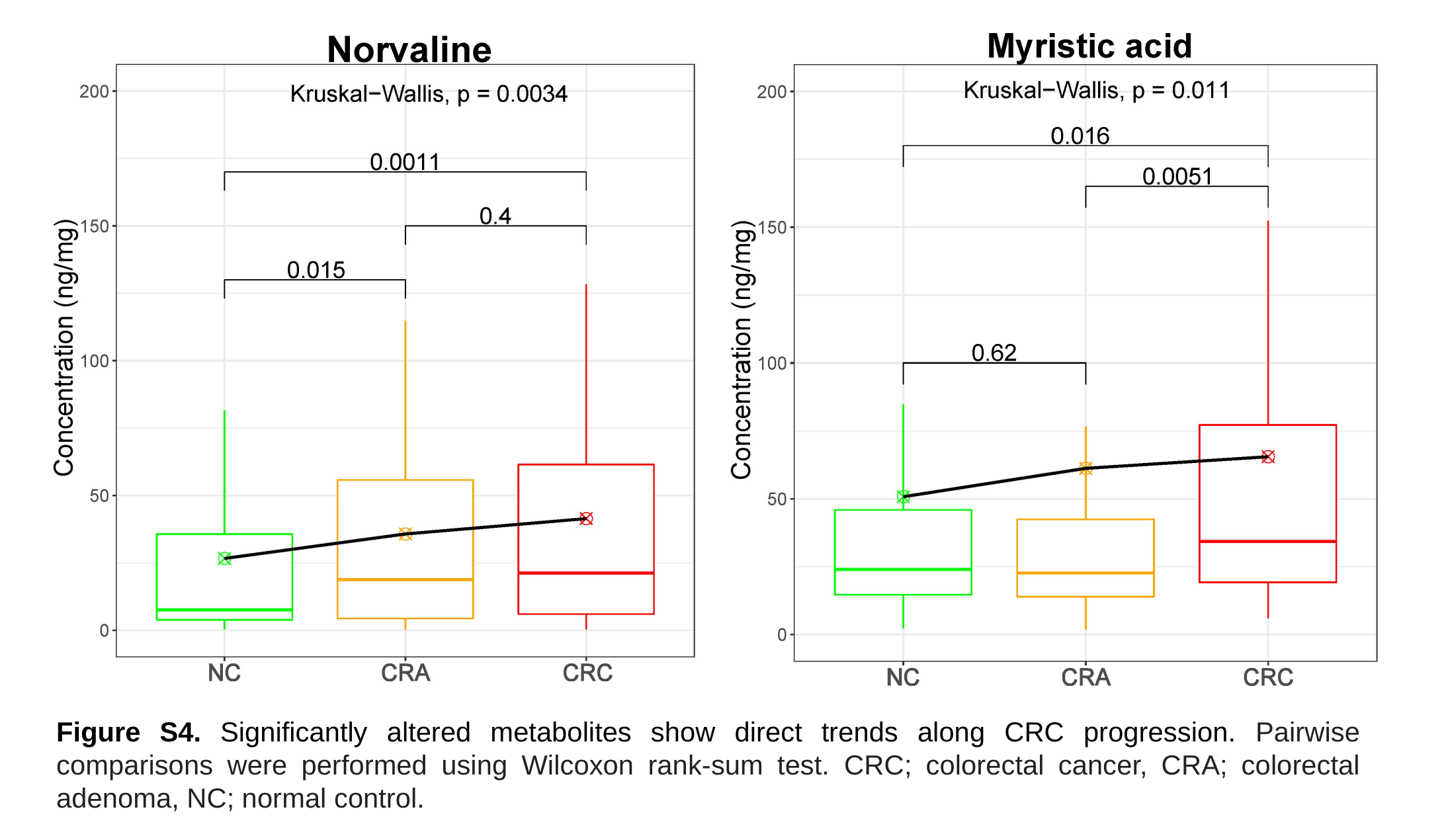

Figure S4. Significantly altered metabolites show direct trends along CRC progression. Pairwise comparisons were performed using Wilcoxon rank-sum test. CRC; colorectal cancer, CRA; colorectal adenoma, NC; normal control.
