# Supplementary material for: Altered gut metabolites and microbiota interactions are implicated in colorectal carcinogenesis and can be non-invasive diagnostic biomarkers
Source: Microbiome. 2022 Feb 21;10:35. doi: 10.1186/s40168-021-01208-5 (PMC8862353; doi:10.1186/s40168-021-01208-5)
Supplement: Supplementary file 11 — Additional file 10: Figure S5. Metabolites markers for pairwise discriminations of CRC, CRA and NC groups with adjustment of age, gender and obesity. [file 40168_2021_1208_MOESM11_ESM.pptx]

## Slide 1
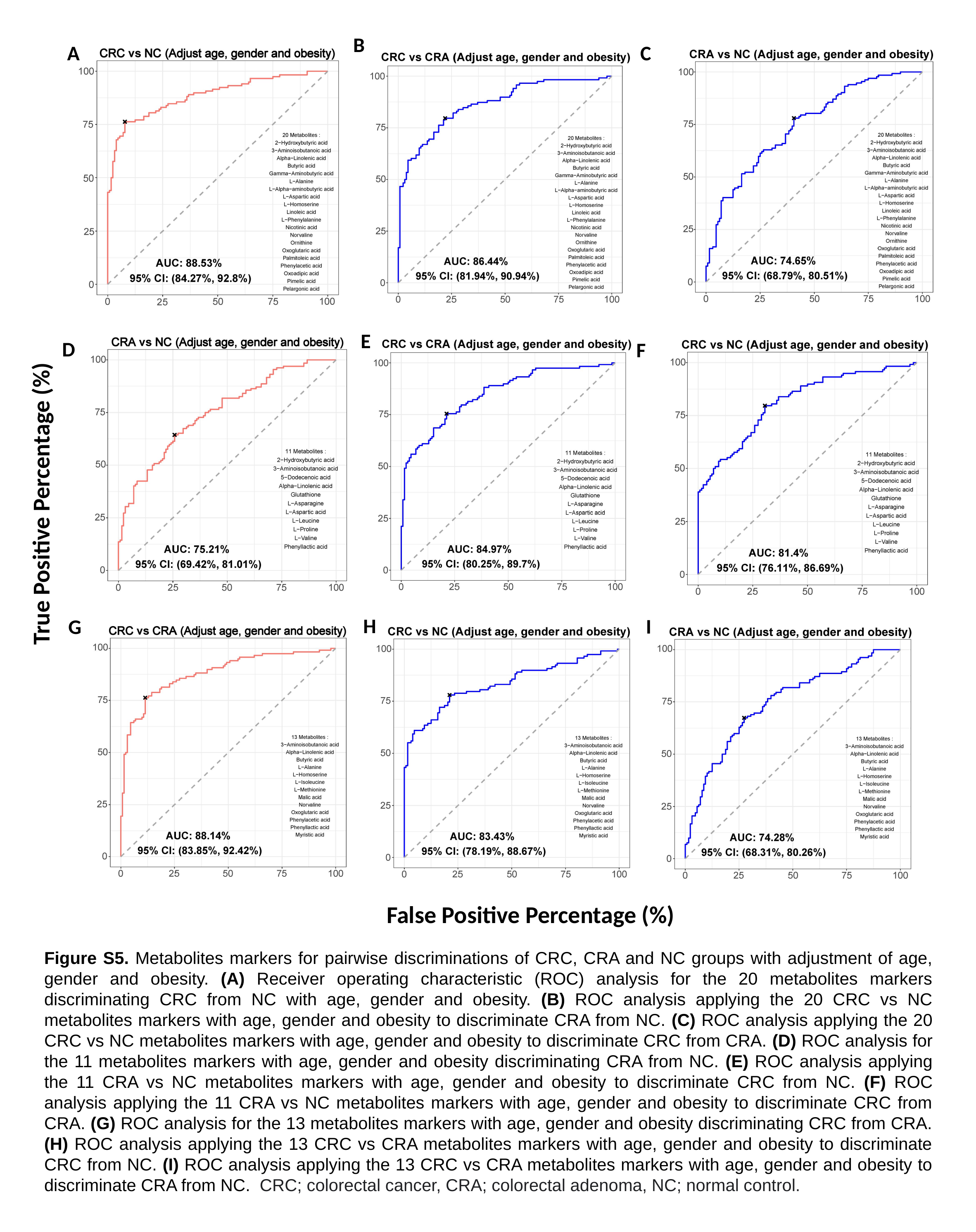

B
A
C
E
D
F
True Positive Percentage (%)
H
I
G
False Positive Percentage (%)
Figure S5. Metabolites markers for pairwise discriminations of CRC, CRA and NC groups with adjustment of age, gender and obesity. (A) Receiver operating characteristic (ROC) analysis for the 20 metabolites markers discriminating CRC from NC with age, gender and obesity. (B) ROC analysis applying the 20 CRC vs NC metabolites markers with age, gender and obesity to discriminate CRA from NC. (C) ROC analysis applying the 20 CRC vs NC metabolites markers with age, gender and obesity to discriminate CRC from CRA. (D) ROC analysis for the 11 metabolites markers with age, gender and obesity discriminating CRA from NC. (E) ROC analysis applying the 11 CRA vs NC metabolites markers with age, gender and obesity to discriminate CRC from NC. (F) ROC analysis applying the 11 CRA vs NC metabolites markers with age, gender and obesity to discriminate CRC from CRA. (G) ROC analysis for the 13 metabolites markers with age, gender and obesity discriminating CRC from CRA. (H) ROC analysis applying the 13 CRC vs CRA metabolites markers with age, gender and obesity to discriminate CRC from NC. (I) ROC analysis applying the 13 CRC vs CRA metabolites markers with age, gender and obesity to discriminate CRA from NC. CRC; colorectal cancer, CRA; colorectal adenoma, NC; normal control.
