# Supplementary material for: Altered gut metabolites and microbiota interactions are implicated in colorectal carcinogenesis and can be non-invasive diagnostic biomarkers
Source: Microbiome. 2022 Feb 21;10:35. doi: 10.1186/s40168-021-01208-5 (PMC8862353; doi:10.1186/s40168-021-01208-5)
Supplement: Supplementary file 12 — Additional file 11: Figure S6. Validation of metabolites markers for pairwise discriminations of CRC, CRA and NC groups by random forest model with 10-fold cross validation. [file 40168_2021_1208_MOESM12_ESM.pptx]

## Slide 1
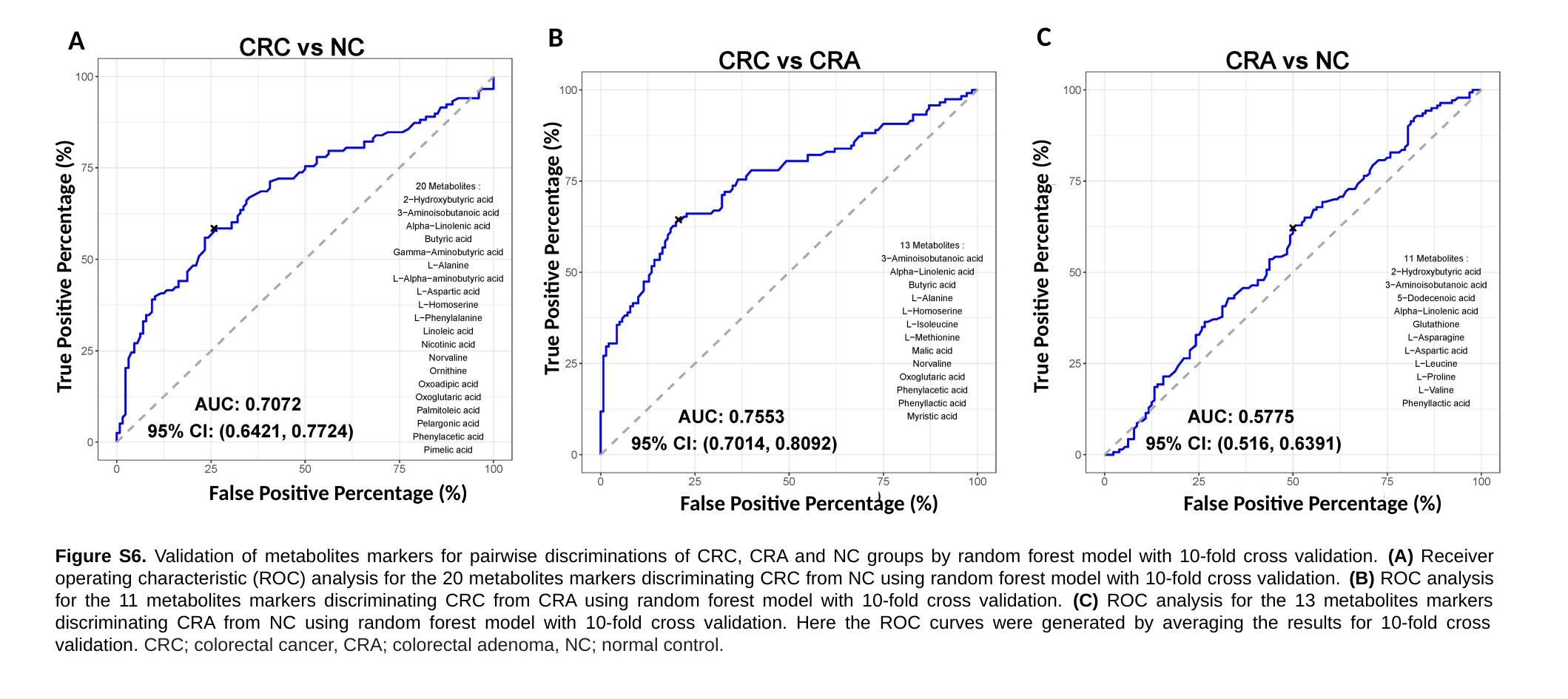

C
B
A
True Positive Percentage (%)
True Positive Percentage (%)
True Positive Percentage (%)
False Positive Percentage (%)
False Positive Percentage (%)
False Positive Percentage (%)
Figure S6. Validation of metabolites markers for pairwise discriminations of CRC, CRA and NC groups by random forest model with 10-fold cross validation. (A) Receiver operating characteristic (ROC) analysis for the 20 metabolites markers discriminating CRC from NC using random forest model with 10-fold cross validation. (B) ROC analysis for the 11 metabolites markers discriminating CRC from CRA using random forest model with 10-fold cross validation. (C) ROC analysis for the 13 metabolites markers discriminating CRA from NC using random forest model with 10-fold cross validation. Here the ROC curves were generated by averaging the results for 10-fold cross validation. CRC; colorectal cancer, CRA; colorectal adenoma, NC; normal control.
