# Supplementary material for: Altered gut metabolites and microbiota interactions are implicated in colorectal carcinogenesis and can be non-invasive diagnostic biomarkers
Source: Microbiome. 2022 Feb 21;10:35. doi: 10.1186/s40168-021-01208-5 (PMC8862353; doi:10.1186/s40168-021-01208-5)
Supplement: Supplementary file 13 — Additional file 12: Figure S7. Differentially abundant bacterial species show direct trends along CRC progression. [file 40168_2021_1208_MOESM13_ESM.pptx]

## Slide 1
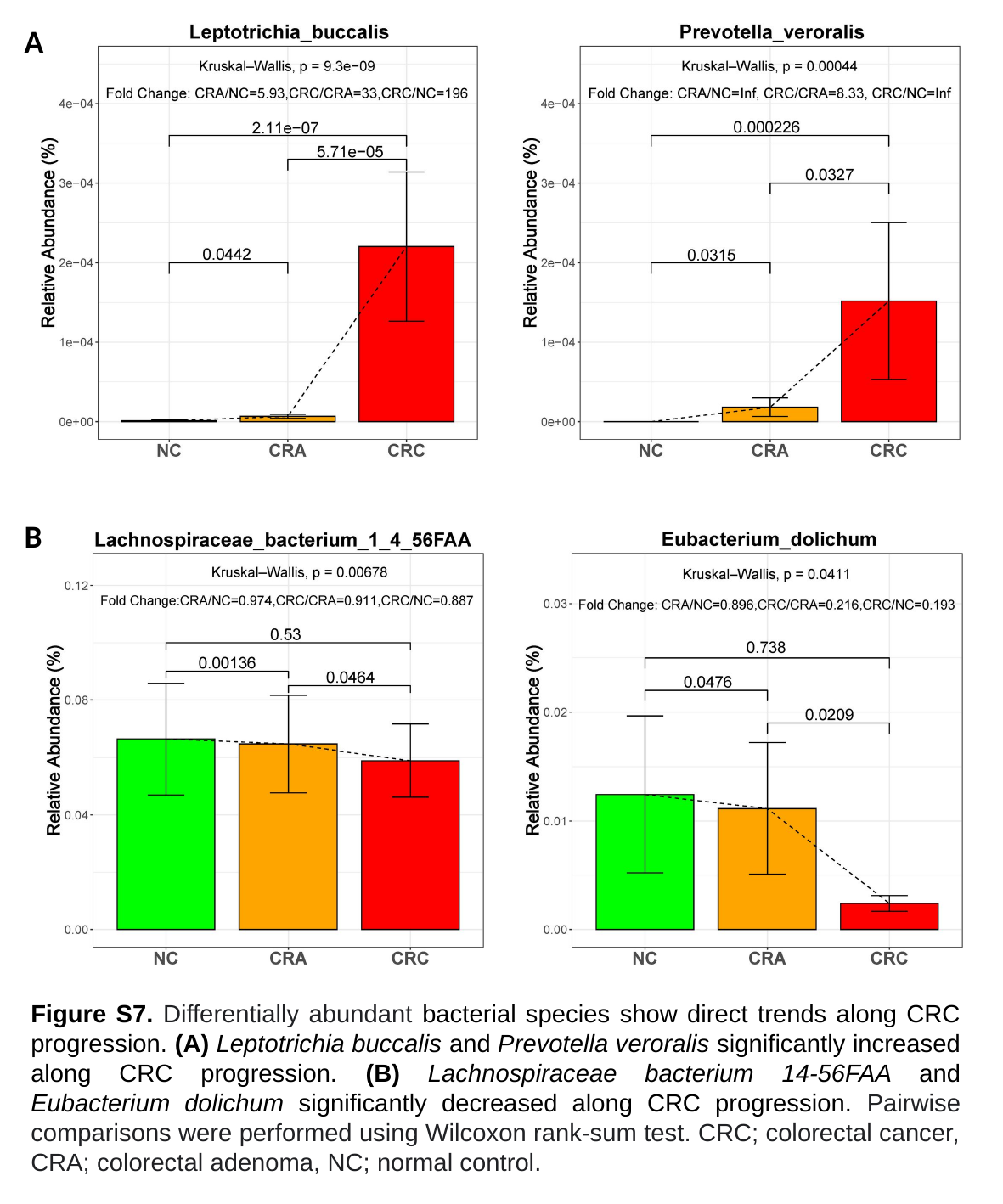

A
B
Figure S7. Differentially abundant bacterial species show direct trends along CRC progression. (A) Leptotrichia buccalis and Prevotella veroralis significantly increased along CRC progression. (B) Lachnospiraceae bacterium 14-56FAA and Eubacterium dolichum significantly decreased along CRC progression. Pairwise comparisons were performed using Wilcoxon rank-sum test. CRC; colorectal cancer, CRA; colorectal adenoma, NC; normal control.
