# Supplementary material for: Altered gut metabolites and microbiota interactions are implicated in colorectal carcinogenesis and can be non-invasive diagnostic biomarkers
Source: Microbiome. 2022 Feb 21;10:35. doi: 10.1186/s40168-021-01208-5 (PMC8862353; doi:10.1186/s40168-021-01208-5)
Supplement: Supplementary file 14 — Additional file 13: Figure S8. Bacterial species markers for pairwise discriminations of CRC, CRA and NC groups. [file 40168_2021_1208_MOESM14_ESM.pptx]

## Slide 1
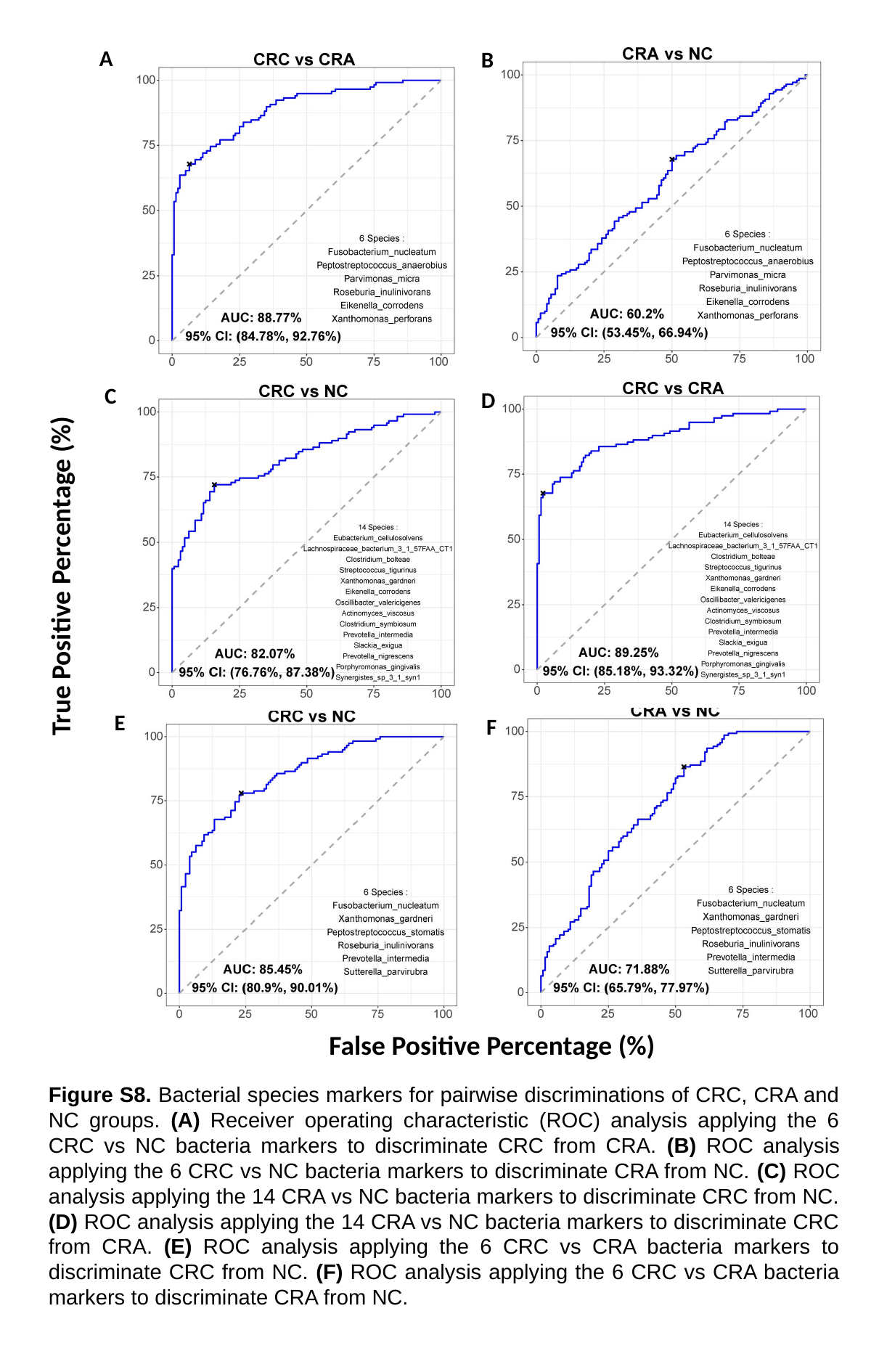

A
B
C
D
True Positive Percentage (%)
E
F
False Positive Percentage (%)
Figure S8. Bacterial species markers for pairwise discriminations of CRC, CRA and NC groups. (A) Receiver operating characteristic (ROC) analysis applying the 6 CRC vs NC bacteria markers to discriminate CRC from CRA. (B) ROC analysis applying the 6 CRC vs NC bacteria markers to discriminate CRA from NC. (C) ROC analysis applying the 14 CRA vs NC bacteria markers to discriminate CRC from NC. (D) ROC analysis applying the 14 CRA vs NC bacteria markers to discriminate CRC from CRA. (E) ROC analysis applying the 6 CRC vs CRA bacteria markers to discriminate CRC from NC. (F) ROC analysis applying the 6 CRC vs CRA bacteria markers to discriminate CRA from NC.
