# Supplementary material for: Altered gut metabolites and microbiota interactions are implicated in colorectal carcinogenesis and can be non-invasive diagnostic biomarkers
Source: Microbiome. 2022 Feb 21;10:35. doi: 10.1186/s40168-021-01208-5 (PMC8862353; doi:10.1186/s40168-021-01208-5)
Supplement: Supplementary file 15 — Additional file 14: Figure S9. Bacterial species markers for pairwise discriminations of CRC, CRA and NC groups with adjustment of age, gender and obesity. [file 40168_2021_1208_MOESM15_ESM.pptx]

## Slide 1
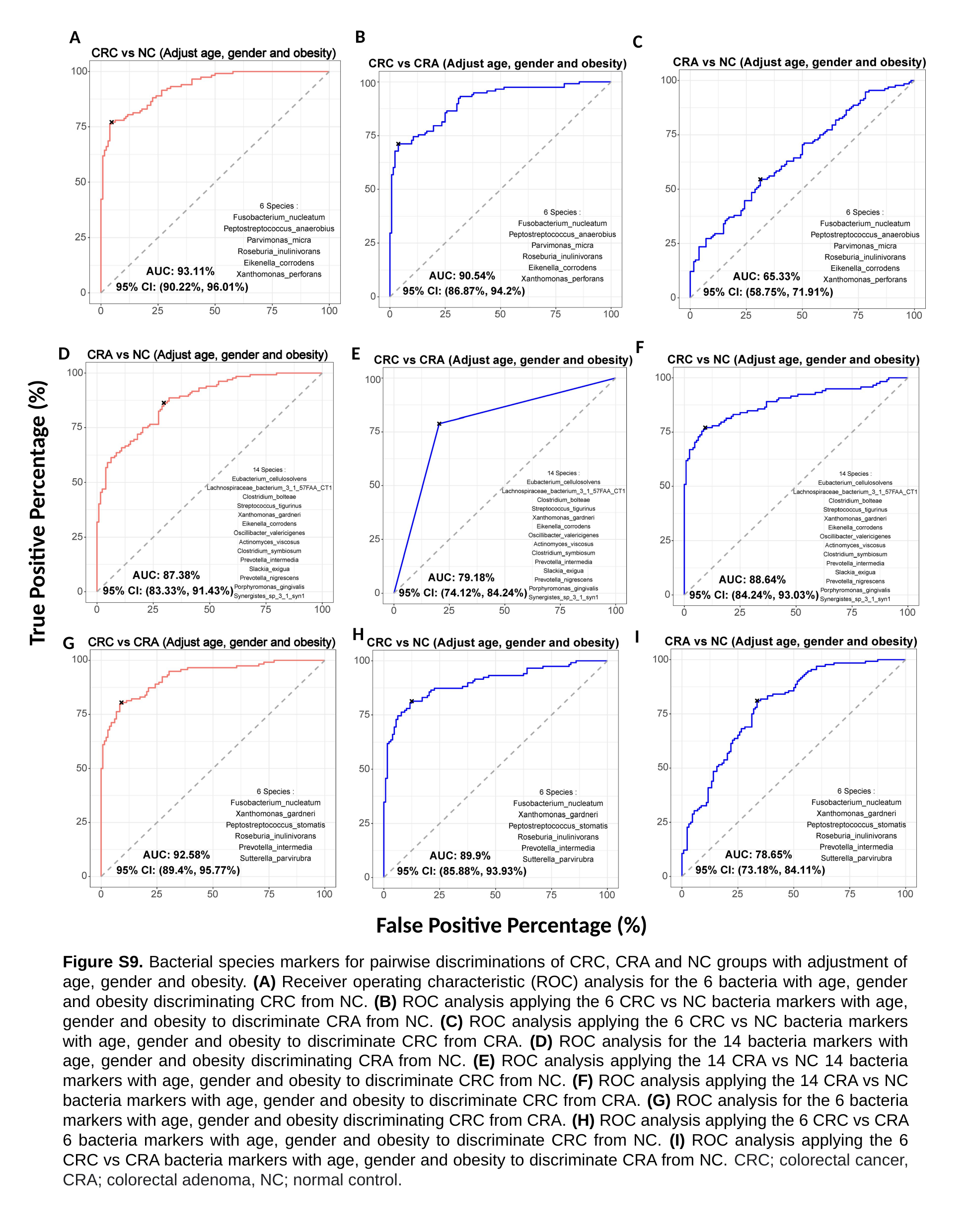

B
A
C
F
E
D
True Positive Percentage (%)
H
I
G
False Positive Percentage (%)
Figure S9. Bacterial species markers for pairwise discriminations of CRC, CRA and NC groups with adjustment of age, gender and obesity. (A) Receiver operating characteristic (ROC) analysis for the 6 bacteria with age, gender and obesity discriminating CRC from NC. (B) ROC analysis applying the 6 CRC vs NC bacteria markers with age, gender and obesity to discriminate CRA from NC. (C) ROC analysis applying the 6 CRC vs NC bacteria markers with age, gender and obesity to discriminate CRC from CRA. (D) ROC analysis for the 14 bacteria markers with age, gender and obesity discriminating CRA from NC. (E) ROC analysis applying the 14 CRA vs NC 14 bacteria markers with age, gender and obesity to discriminate CRC from NC. (F) ROC analysis applying the 14 CRA vs NC bacteria markers with age, gender and obesity to discriminate CRC from CRA. (G) ROC analysis for the 6 bacteria markers with age, gender and obesity discriminating CRC from CRA. (H) ROC analysis applying the 6 CRC vs CRA 6 bacteria markers with age, gender and obesity to discriminate CRC from NC. (I) ROC analysis applying the 6 CRC vs CRA bacteria markers with age, gender and obesity to discriminate CRA from NC. CRC; colorectal cancer, CRA; colorectal adenoma, NC; normal control.
