# Supplementary material for: Altered gut metabolites and microbiota interactions are implicated in colorectal carcinogenesis and can be non-invasive diagnostic biomarkers
Source: Microbiome. 2022 Feb 21;10:35. doi: 10.1186/s40168-021-01208-5 (PMC8862353; doi:10.1186/s40168-021-01208-5)
Supplement: Supplementary file 17 — Additional file 16: Figure S11. Combination of bacteria and metabolites markers for pairwise discriminations of CRC, CRA and NC groups. [file 40168_2021_1208_MOESM17_ESM.pptx]

## Slide 1
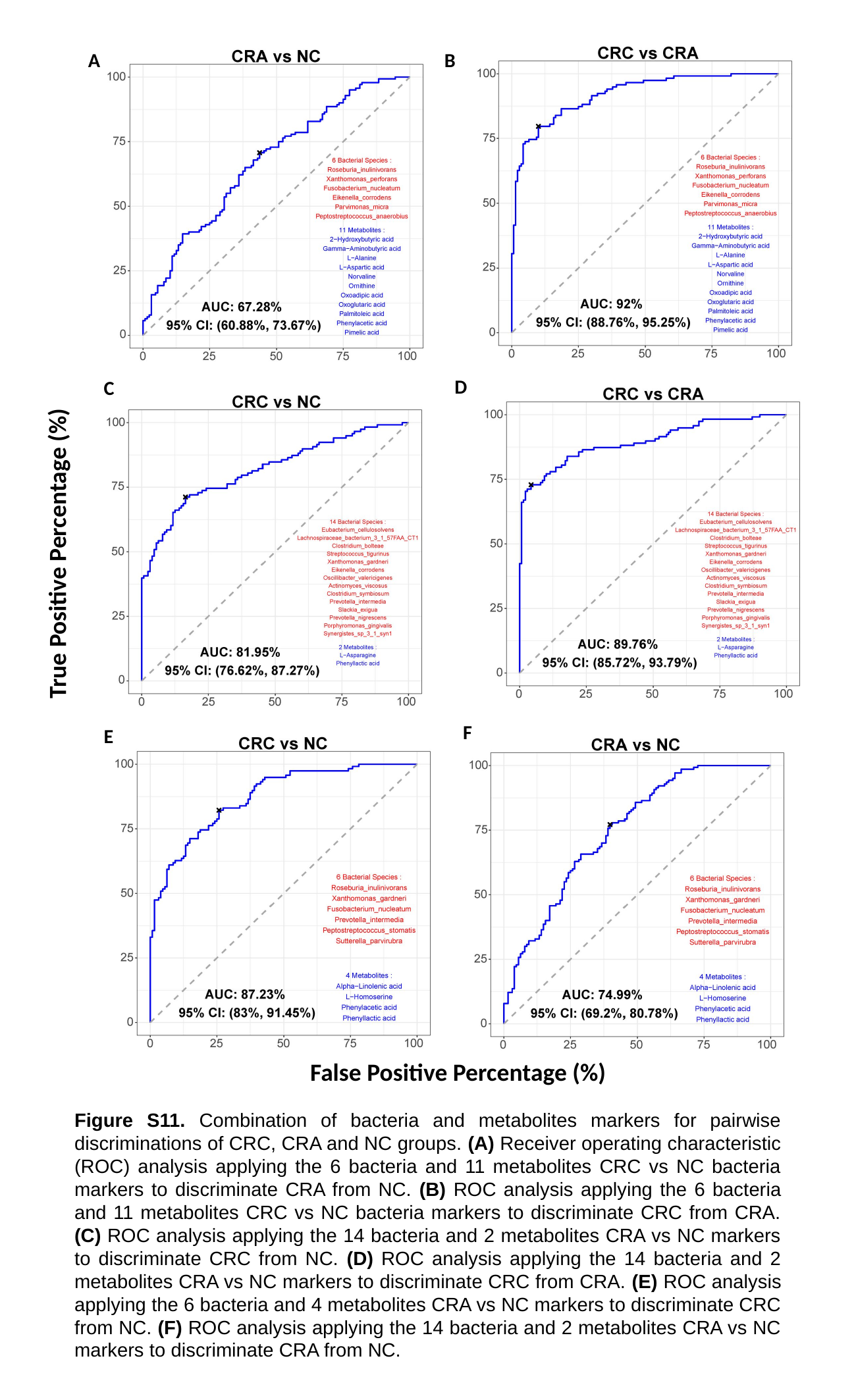

A
B
D
C
True Positive Percentage (%)
F
E
False Positive Percentage (%)
Figure S11. Combination of bacteria and metabolites markers for pairwise discriminations of CRC, CRA and NC groups. (A) Receiver operating characteristic (ROC) analysis applying the 6 bacteria and 11 metabolites CRC vs NC bacteria markers to discriminate CRA from NC. (B) ROC analysis applying the 6 bacteria and 11 metabolites CRC vs NC bacteria markers to discriminate CRC from CRA. (C) ROC analysis applying the 14 bacteria and 2 metabolites CRA vs NC markers to discriminate CRC from NC. (D) ROC analysis applying the 14 bacteria and 2 metabolites CRA vs NC markers to discriminate CRC from CRA. (E) ROC analysis applying the 6 bacteria and 4 metabolites CRA vs NC markers to discriminate CRC from NC. (F) ROC analysis applying the 14 bacteria and 2 metabolites CRA vs NC markers to discriminate CRA from NC.
