# Supplementary material for: Altered gut metabolites and microbiota interactions are implicated in colorectal carcinogenesis and can be non-invasive diagnostic biomarkers
Source: Microbiome. 2022 Feb 21;10:35. doi: 10.1186/s40168-021-01208-5 (PMC8862353; doi:10.1186/s40168-021-01208-5)
Supplement: Supplementary file 18 — Additional file 17: Figure S12. Distributions of association between significantly altered metabolites and bacterial species for CRC, CRA and NC groups. [file 40168_2021_1208_MOESM18_ESM.pptx]

## Slide 1
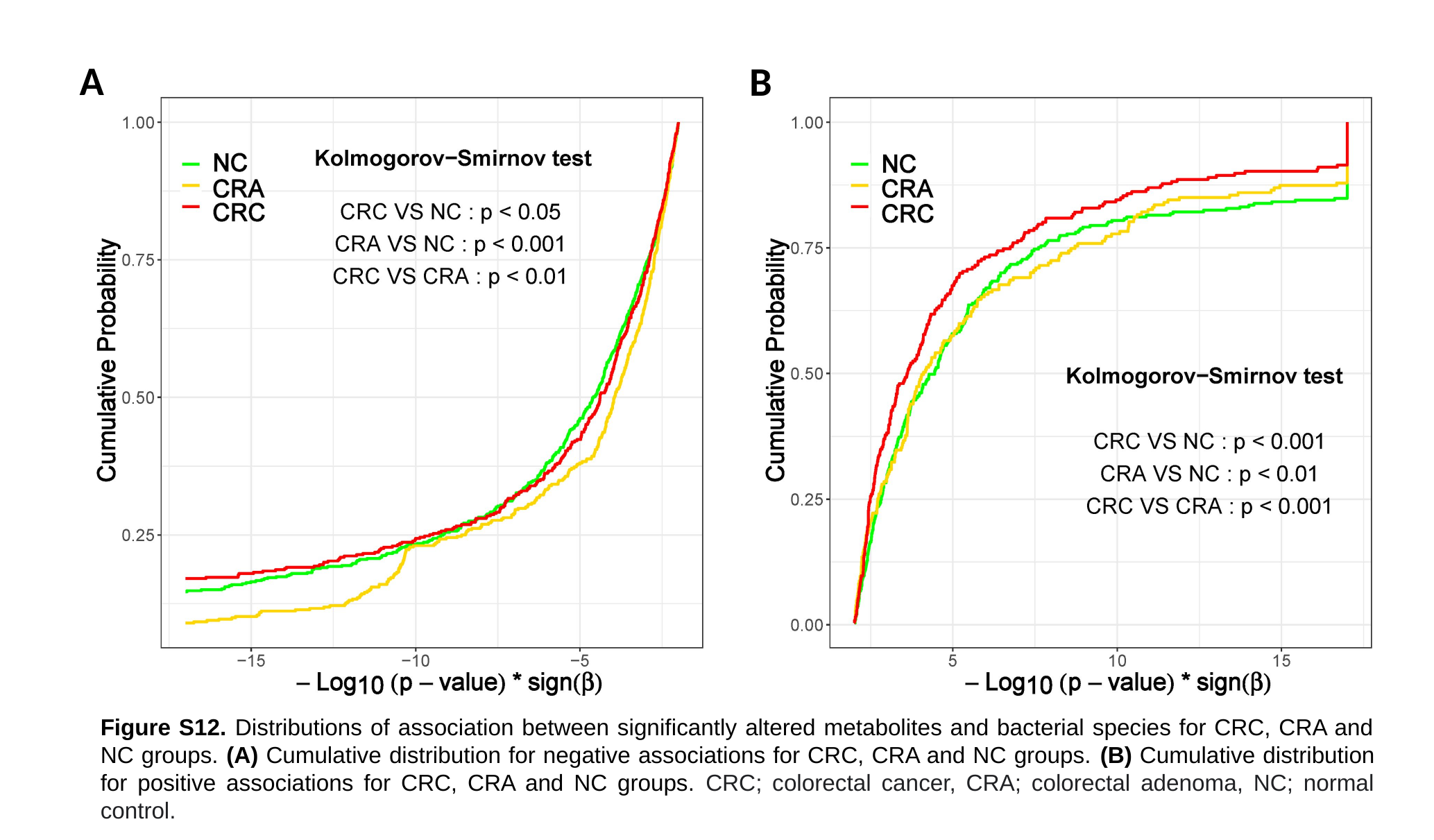

A
B
Figure S12. Distributions of association between significantly altered metabolites and bacterial species for CRC, CRA and NC groups. (A) Cumulative distribution for negative associations for CRC, CRA and NC groups. (B) Cumulative distribution for positive associations for CRC, CRA and NC groups. CRC; colorectal cancer, CRA; colorectal adenoma, NC; normal control.
