# Supplementary material for: Altered gut metabolites and microbiota interactions are implicated in colorectal carcinogenesis and can be non-invasive diagnostic biomarkers
Source: Microbiome. 2022 Feb 21;10:35. doi: 10.1186/s40168-021-01208-5 (PMC8862353; doi:10.1186/s40168-021-01208-5)
Supplement: Supplementary file 19 — Additional file 18: Figure S13. Interactions among metabolites and bacteria are altered in CRC. [file 40168_2021_1208_MOESM19_ESM.pptx]

## Slide 1
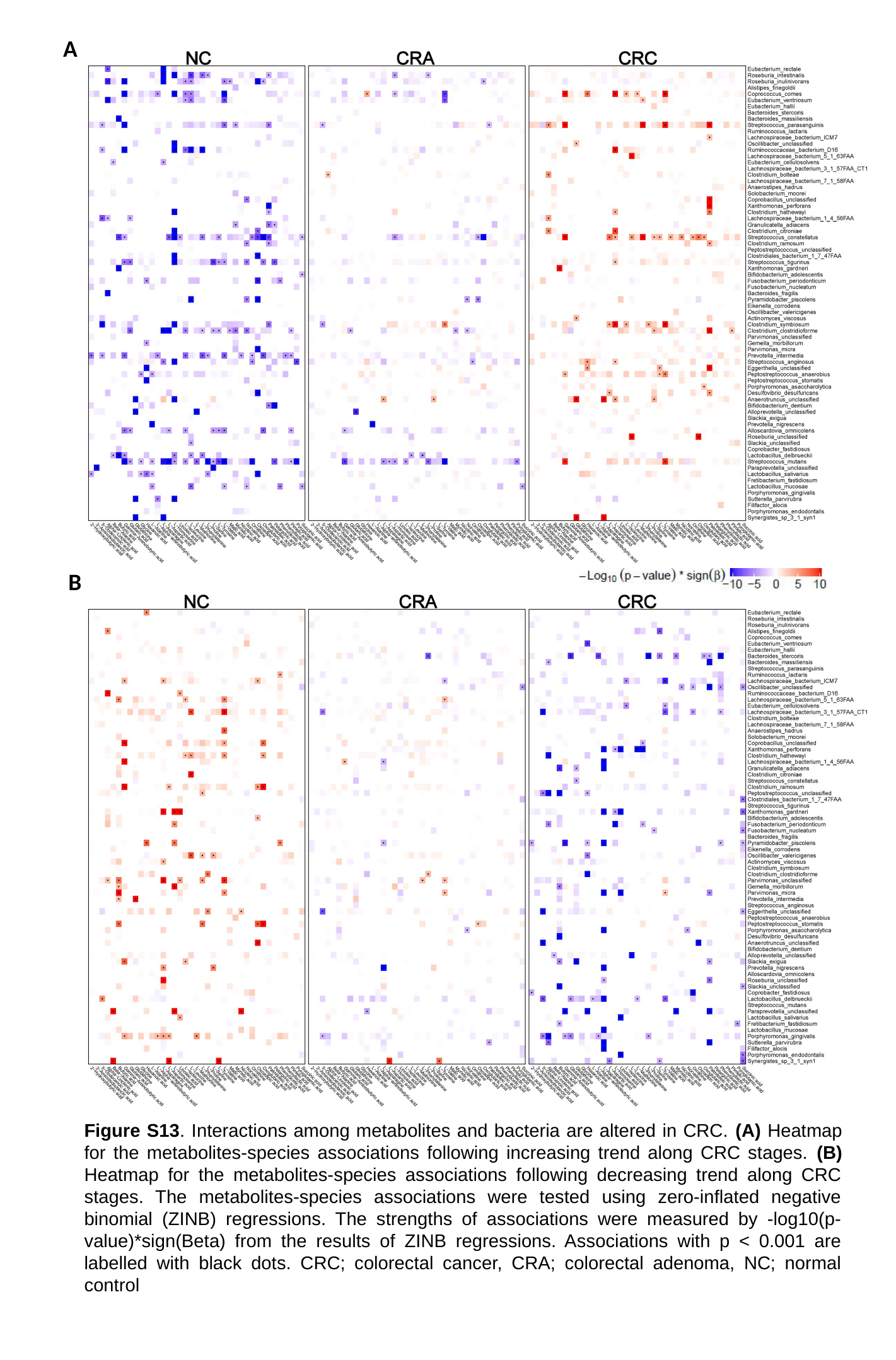

A
B
Figure S13. Interactions among metabolites and bacteria are altered in CRC. (A) Heatmap for the metabolites-species associations following increasing trend along CRC stages. (B) Heatmap for the metabolites-species associations following decreasing trend along CRC stages. The metabolites-species associations were tested using zero-inflated negative binomial (ZINB) regressions. The strengths of associations were measured by -log10(p-value)*sign(Beta) from the results of ZINB regressions. Associations with p < 0.001 are labelled with black dots. CRC; colorectal cancer, CRA; colorectal adenoma, NC; normal control
