# Supplementary material for: Altered gut metabolites and microbiota interactions are implicated in colorectal carcinogenesis and can be non-invasive diagnostic biomarkers
Source: Microbiome. 2022 Feb 21;10:35. doi: 10.1186/s40168-021-01208-5 (PMC8862353; doi:10.1186/s40168-021-01208-5)
Supplement: Supplementary file 20 — Additional file 19: Figure S14. Heatmap of correlations between disease associated metabolites. The correlation strengths were measured by Spearman’s rank correlation coefficient. Only correlation coefficients with p > 0.05 were shown on the heatmap. The size of the circles are proportional to the correlation strength. [file 40168_2021_1208_MOESM20_ESM.pptx]

## Slide 1
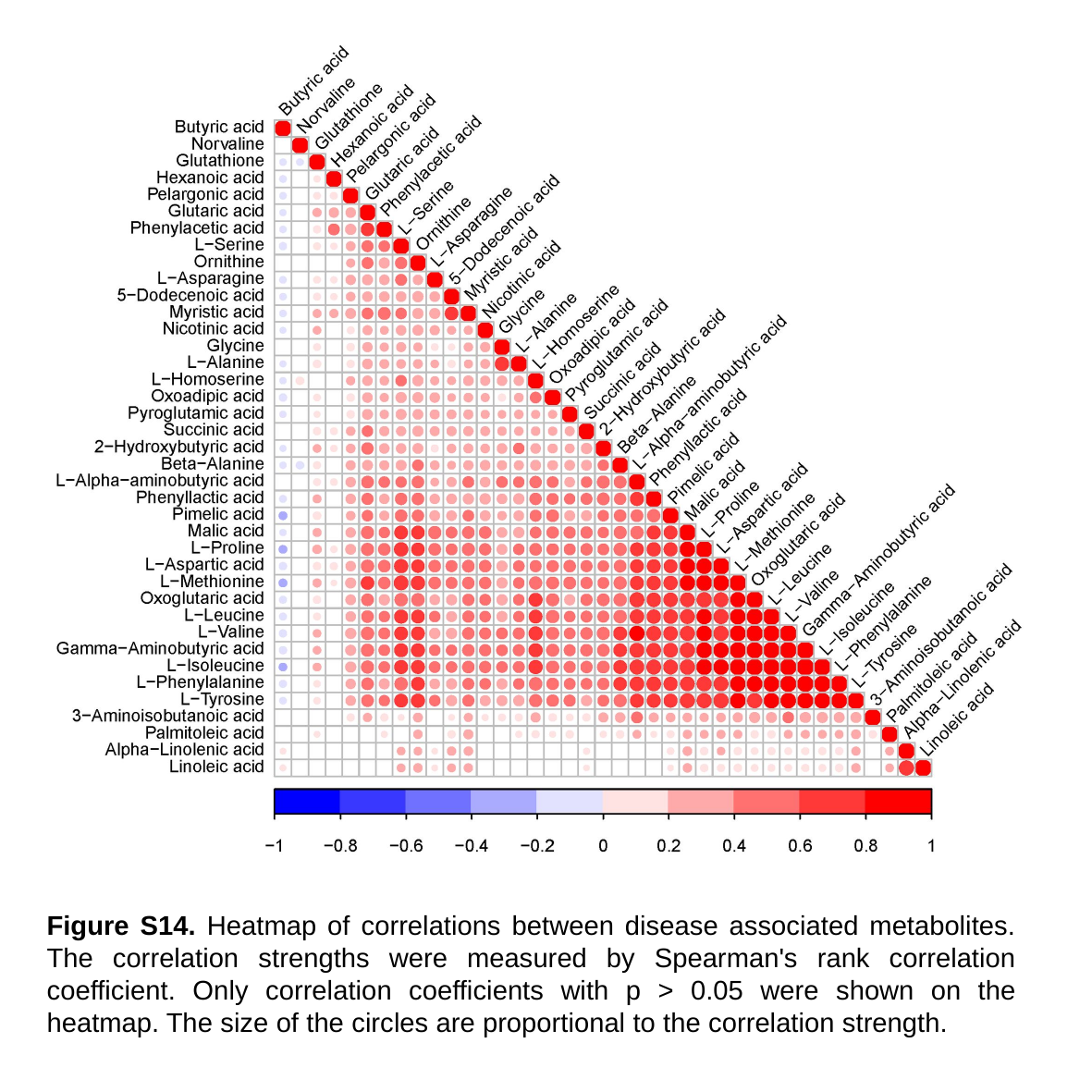

Figure S14. Heatmap of correlations between disease associated metabolites. The correlation strengths were measured by Spearman's rank correlation coefficient. Only correlation coefficients with p > 0.05 were shown on the heatmap. The size of the circles are proportional to the correlation strength.
